# Miramides A–D: Identification of Detoxin-like Depsipeptides after Heterologous Expression of a Hybrid NRPS-PKS Gene Cluster from *Streptomyces mirabilis* Lu17588

**DOI:** 10.3390/microorganisms10091752

**Published:** 2022-08-30

**Authors:** Constanze Paulus, Maksym Myronovskyi, Josef Zapp, Marta Rodríguez Estévez, Maria Lopatniuk, Birgit Rosenkränzer, Anja Palusczak, Andriy Luzhetskyy

**Affiliations:** 1 Department of Pharmaceutical Biotechnology, Saarland University, 66123 Saarbruecken, Germany; 2 Department of Pharmaceutical Biology, Saarland University, 66123 Saarbruecken, Germany; 3 AMEG Department, Helmholtz Institute for Pharmaceutical Research Saarland, 66123 Saarbruecken, Germany

**Keywords:** *Streptomyces*, natural products, heterologous expression, hybrid NRPS-PKS

## Abstract

Natural products derived from plants, fungi or bacteria have been used for years in the medicine, agriculture and food industries as they exhibit a variety of beneficial properties, such as antibiotic, antifungal, anticancer, herbicidal and immunosuppressive activities. Compared to synthetic compounds, natural products possess a greater chemical diversity, which is a reason why they are profitable templates for developing pharmaceutical drug candidates and ongoing research on them is inevitable. Performing heterologous expression with unknown gene clusters is the preferred method to activate gene clusters that are not expressed in the wild-type strain under laboratory conditions; thus, this method offers a way to discover new interesting metabolites. Here, we report the gene cluster assembly of a hybrid NRPS-PKS gene cluster from *Streptomyces mirabilis* Lu17588, which was heterologously expressed in *Streptomyces albus* Del14. Four new compounds were produced by the obtained strain, which were named miramides A–D. Isolation and structure elucidation revealed similarity of the isolated compounds to the known depsipeptides rimosamides/detoxins.

## 1. Introduction

Exploring the vast array of bacterial strains allowed for the discovery of many valuable natural products that are applicable in different areas of life [1]. However, accessing the natural products encoded in the chromosome of the producing strain is not always straightforward. Due to the low or even missing expression of biosynthetic gene clusters (BGCs) under laboratory conditions, the encoded natural products are only sparsely available in the culture broth and thus are difficult to isolate in sufficient amounts [2,3]. For instance, the genus *Streptomyces* usually harbors approximately 30 BGCs per genome, but only 10% are efficiently expressed under standard cultivation conditions, meaning that 90% remain unexplored if the strains are examined with conventional methods [4]. To obtain access to the metabolites encoded by the silent gene clusters, various methods have been developed over the years.

Activation of the silent gene clusters in the native host strains is a widely used approach to discover new secondary metabolites. To activate the production of natural products with this strategy, different target genes could be manipulated, such as various pleiotropic regulatory genes that can be scattered over the genome and affect secondary metabolism globally or cluster-situated regulators that mostly regulate the expression of a particular pathway. Depending on whether the regulator exerts a positive or negative effect on the expression of the pathway, the corresponding encoding gene can be either overexpressed or deleted. For instance, the production of chattamycin B was achieved through the overexpression of a pathway-specific regulator under control of the strong constitutive ermE* promoter in the native strain [5]. Another widely applied approach to activate indigenous clusters is ribosome and RNA-polymerase engineering [6]. Point mutations that often confer resistance to antibiotics are introduced either into the *rpsL* gene encoding S12 ribosomal protein or into the *rpoB* gene encoding the β subunit of RNA polymerase. The missense H437Y mutation in the *rpoB* gene led to the expression of the anthrachamycin gene cluster in *S. chattanoogensis* L10 [7]. The OSMAC approach to activate internal gene clusters is a method that utilizes the changing metabolite production that occurs through varying cultivation conditions, such as the medium composition, temperature, pH, oxygen level or addition of enzymes/salts/amino acids. In this way, the production of surugamide A in *Streptomyces* sp. SM17 was significantly increased, namely, to a 10-fold higher level, when the medium was changed from SYP-NaCl to YD medium [8]. Recently, the development of high-throughput elicitor screening (HITES) for the activation of silent gene clusters was reported [9]. Here, the examined strain was cultivated in a high-throughput manner with a library of chemical compounds. The successful activation of a cluster can be detected with the help of a reporter gene that was previously inserted into the cluster, by bioactivity screens or MALDI mass spectrometry [10]. The HITES approach was successfully applied for the activation of taylorflavin A production in *Streptomyces hiroshimensis* and cinnapeptin production in *Streptomyces ghanaensis* [9,11]. Strategies to directly activate natural product biosynthetic pathways in natural producers have several drawbacks. Many of the previously described approaches are undirected, meaning that they cannot be applied for the direct activation of a particular gene cluster within the chromosome. It is impossible to foresee which of the encoded silent clusters will be activated by ribosome or RNA polymerase engineering, the OSMAC approach or manipulation of pleiotropic genes and whether any effect will occur. Furthermore, to manipulate regulatory genes, established methods must be applied to transfer DNA into the producing strains. Considering that many of the natural producers are refractory to genetic manipulations, the success of this cluster activation approach is not guaranteed.

Another complementary strategy to activate biosynthetic pathways is their expression in surrogate host strains. For this purpose, the BGCs of interest are cloned into an *E. coli*-*Streptomyes* shuttle vector and transferred into one or several heterologous hosts. The host strain is commonly adapted for expressing natural products, meaning that the strain usually has a fast growth rate, is genetically tractable and is optimized through deleting indigenous pathways that encode the production of background metabolites [12,13]. Deleting the secondary metabolite clusters reduces the metabolic burden of the strain and therefore increases the concentration of the most common intracellular biosynthetic precursors, which in turn may facilitate the production of compounds of interest. Therefore, heterologously expressed pathways often lead to production rates that are higher than those in native organisms. For instance, the production yields of bafilomycin B1, lactacystin, holomycin, pholipomycin and chloramphenicol were much higher in the genome-minimized heterologous host *Streptomyces avermitilis* SUKA22 than in the respective original producer strains [14]. The detection and purification of biosynthetic products is further simplified by the reduced metabolic background of the heterologous host. For cases in which the heterologous expression of the intact cluster does not lead to the activation of compound production or if the production levels are not sufficient for compound isolation, the pathway can be refactored in *E. coli,* and the resulting optimized construct can be expressed in the heterologous host again. The heterologous expression strategy is straightforward and does not involve the drawbacks of manipulating naturally producing strains. However, constructing and sequencing the genomic library, which is used as a source of clusters for heterologous expression, is laborious and time-consuming. Furthermore, the borders of a BGC cannot be predicted reliably, which might prevent successful heterologous expression. Additionally, in some cases, resistance issues involving the host strain may hinder the success of heterologous expression, too. Currently, several optimized *Streptomyces* host strains are available for heterologous expression of natural product BGCs. Among the most commonly used hosts are *S. coelicolor* M1152, *S. coelicolor* M1154, *S. albus* Del14, *S. lividans* TK24, *S. avermitilis* SUKA 17 and *S. avermitilis* pKU462 [13,14,15,16].

In this paper, we report the assembly and heterologous expression of a hybrid NRPS-PKS gene cluster from *Streptomyces mirabilis* Lu 17588 in *S. albus* Del14. Four new metabolites were detected as a result of cluster expression. Isolation and structure elucidation of the detected compounds revealed that the compounds are new depsipeptides with core structures similar to those of the known blasticidin antagonists detoxins and rimosamides. The detoxin complex was previously isolated from *S. caespitosus* var. *detoxicus* 7072 GC_1_ by Kakinuma et al. in 1974, and this complex consisted of a number of compounds that shared the core structure Val/Ile-Det-Phe and an alternating fatty acid chain attached to Phe. The rimosamides were isolated from *S*. *rimosus* NRRL B-2659 by McClure et al. in 2016. They share the same core structure but instead of a fatty acid part, they possess an isobutyrate-glycyl (Ibu-Gly) moiety [17,18]. The peptide core of the isolated products is the same as in detoxins/rimosamides; however, a short hyrdoxylated fatty acid side chain was found attached to Phe for the products of the heterologously expressed hybrid NRPS-PKS BGC. This moiety is new and differs in chain length for each derivative. The four compounds were unambiguously identified and named miramides A–D (**1**–**4**).

## 2. Materials and Methods

### 2.1. General Experimental Procedures

*Streptomyces* strains were grown on solid MS (mannitol soy flour; mannitol 20 g/L, soy 20 g/L) medium and in liquid TSB (tryptic soy broth, Sigma-Aldrich, St. Louis, MO, USA) medium [19]. *E. coli* strains were grown in solid or liquid LB (lysogeny broth) medium [20]. Liquid DNPM medium (dextrin 40 g/L, bacto soytone 7.5 g/L, yeast extract 5 g/L and MOPS (4-morpholinepropanesulfonic acid) 21 g/L, pH 6.8) was used as the medium for secondary metabolite production. The *E. coli* strains GB05redec and ET 12567 pUB 307 were used for cosmid assembly and cloning (Table S1). When needed, the antibiotic apramycin (50 µg/mL; Sigma-Aldrich, St. Louis, MO, USA) and nalidixic acid (50 µg/mL; Carl Roth, Karlsruhe, Germany) were added to the medium.

### 2.2. Cloning and Heterologous Expression of NRPS-PKS Gene Cluster

For the assembly of the NRPS-PKS cluster, the cosmids 11_E04, 06_C04 and 08_G05 containing overlapping chromosome fragments of *S. mirabilis* were selected from the previously constructed genomic library of the strain. The genomic library was end-sequenced, which allows the cloned fragment of each single cosmid to be precisely identified. The cosmid library of *S. mirabilis* was constructed on a cos15A_gus backbone (GenBank accession number: OL702928), which cannot be propagated in *Streptomyces* strains (Table S1). To express the cloned fragment in *Streptomyces* hosts, it should be recloned into the integrative pSMART_assembl BAC (GenBank accession number: OM108702), which is then transferred into *Streptomyces* strains (Table S1). This vector contains phage *phi*C31 integrase and *att*P sites as well as a selection marker for streptomycetes. The TAR assembly is used for the recloning step [21,22]. The vectors cos15A_gus and pSMART_assembl allow for both the direct recloning of a single fragment or assembly of several overlapping fragments. cos15A_gus contains two DNA fragments (homology arms) that flank the cloning site and are identical to the regions within the BAC vector pSMART_assembl. These homology arms enable the chromosomal fragments to be recloned using TAR. With the help of a rare-cutter enzyme, the fragments cloned in cos15A_gus are cut out with either one homology arm, both homology arms or without homology arms. When it is necessary to reclone a single fragment, it is cut with both homology arms, which enables it to be integrated into the BAC vector through homology recombination. When two overlapping fragments need to be assembled and recloned into the BAC vector, the first fragment is cut out with the left homology arm, and the second fragment is cut out with the right homology arm. Recombination between homology arms of the fragments and of the BAC backbone as well as recombination between the overlapping fragments is needed for successful assembly. When three or more overlapping fragments are assembled on the BAC backbone, the outermost left fragment should be cut with the left homology arm, and the outermost right fragment should be cut with the right homology arm. The middle fragments were cut without any homology arms. The recloning scheme is shown in Figure S1.

For assembly, the cosmids 11_E04, 06_C04 and 08_G05 were digested with I-SceI/PacI, I-CeuI/PacI and I-SceI/I-CeuI combinations of restriction endonucleases, respectively (Figure S1). The BAC vector pSMART_assembl was digested with MssI. The digested cosmids and the BAC backbone were precipitated and mixed together. The DNA mixture was transferred into *Saccharomyces cerevisiae* BY4742 using a standard lithium acetate protocol [23]. The recombinants were selected on synthetic complete (SC) medium [24] supplemented with yeast synthetic leucine drop-out medium (Sigma-Aldrich) at a concentration of 2 g/L. *S. cerevisiae* clones containing the assembled construct were identified by colony-PCR. The total DNA of the positive *S. cerevisiae* clones was isolated and transferred into *E. coli* GB05 red by transformation. The assembled BAC pSMART_cl1 was isolated and checked by restriction mapping and PCR.

For heterologous expression, the BAC pSMART_cl1 was transferred into *S. albus* Del14 via conjugation as described previously [16,25].

### 2.3. Cultivation, Metabolite Extraction and Dereplication

The mutant strain *S. albus* pSMART_cl1 was pre-cultivated in 100 mL flasks with 10 mL of TSB medium (tryptic soy broth 30 g/L) at 28 °C and 180 rpm on a rotary shaker for 24 h. The main culture, using 50 mL of DNPM medium (dextrin 40 g/L, bacto soytone 7.5 g/L, yeast extract 5 g/L and MOPS 21 g/L, pH 6.8) in a 500 mL flask, was inoculated with 1 mL of preculture and cultivated for 5 days at 28 °C and 180 rpm on a rotary shaker. After cultivation, biomass was separated from the supernatant, and the liquid was extracted with 25 mL butanol. The solvent was removed until dry using a nitrogen sample concentrator while heating at 45 °C. The dried supernatant was taken up in 300 µL of methanol and subjected to high-resolution mass spectrometry (HRMS) analysis. Liquid chromatography (LC) was performed on a Dionex Ultimate 3000 UHPLC system (Thermo Fisher Scientific, Waltham, MA, USA) with a stationary phase Acquity UPLC BEH C18, 100 mm × 2.1 mm, 1.7 µm column (Waters Corporation, Milford, MA, USA), using a linear gradient from 5% [B] (acetonitrile + 0.1% formic acid) against [A] (ddH_2_O + 0.1% formic acid) to 95% [B] and coupled to a photo diode array (PDA) detector that operated at 200–600 nm. High-resolution mass spectrometry spectra were acquired on a maXIS II™ mass spectrometer (Bruker, Billerica, MA, USA) using positive ionization mode and mass range detection from *m/z* 200 to 2000. For dereplication, the Dictionary of Natural Products (DNP) (CRC Press, Boca Raton, USA), version 28.1, was used as a database.

### 2.4. Genome Mining of S. Mirabilis Lu175588

The genome of *S. mirabilis* Lu17588 was analyzed using the secondary metabolite gene cluster detection tool antiSMASH (https://antismash.secondarymetabolites.org/#!/start, accessed on 5 November 2018) [26]. Geneious software, v. 11.0.3 (Biomatters Ltd, Auckland, New Zealand) was used to analyze the cosmid library and detect the correct chromosomal fragments. The genomic sequence of the gene cluster under examination was deposited in GenBank under accession number OM108703.

### 2.5. Isolation and Purification of Metabolites Identified after Heterologus Expression

To purify the targeted compounds, the mutant strain *S. albus pSMART_cl1* was cultivated in 10 L DNPM as described above. After cultivation, the supernatant was separated from the biomass and extracted twice with butanol using the same amount of butanol as the supernatant. The solvent was removed on a rotary evaporator at 10–20 mbar and 50 °C water bath temperature. The dry supernatant was dissolved in 100 mL methanol and centrifuged, the pellet was discarded, and the supernatant was dried on a rotary evaporator at 10–20 mbar and 50 °C water bath temperature, resulting in 12 g of raw material. The crude extract was dissolved in 20 mL methanol and pre-purified by flash chromatography (Isolera™ One, Biotage, Uppsala, Sweden) using a Chromabond^®^ Flash RS 330 C18 ec 360 g column (Macherey-Nagel, Düren, Germany). Two runs (10 mL of extract for each) were performed using MQ-H_2_O [A]/methanol [B] without additives as eluents. A linear gradient from 30–70% [B] over 5 column volumes (CV) was applied. Fractions were tested on LC-MS, a Dionex Ultimate 3000 UPLC system (Thermo Fisher Scientific, Waltham, MA, USA) using Acquity BEH C18, 50 × 2.1 mm, 1.7 µm d_p_ column (Waters Corporation, Milford, MA, USA) and the following mobile phase: ddH_2_O + 0.1% formic acid [A]/acetonitrile + 0.1% formic acid [B], 5–95% [B] over 3 min, at flow rate 0.6 mL/min, coupled to amaZON SL speed mass spectrometer (Bruker, Billerica, MA, USA) with ESI source and mass range *m/z* 200–2000. Fractions containing miramides (retention time miramide A = 2.4 min; miramide B/C = 2.4, D = 2.3 min) were combined and further purified on a Waters AutoPurification™ system coupled to a single quadrupole mass detector (Waters, Milford, MA, USA). As for the stationary phase, a Nucleodur C18 HTEC, 250 mm × 21 mm, 5 µm column (Macherey-Nagel, Düren, Germany) and water [A]/methanol [B] without additives as eluents were used. A linear gradient from 50% to 95% [B] in 20 min was applied, and fractions containing the targeted compounds were combined. As the last step, the fractions collected from waters purification, which contained the respective compounds, were combined and purified on a semipreparative HPLC (Agilent 1100, Agilent Technologies, Santa Clara, CA, USA) using a Nucleodur C18 HTEC 250 mm × 10 mm, 5 µm column (Macherey-Nagel, Düren, Germany) and MQ-H_2_O [A]/acetonitrile [B] without additives as the eluent. A linear gradient from 5% to 50% over 20 min was applied, and the fractions were tested by LC-MS (see above) to identify the fractions that contained the substance of interest. The fractions with the same compound were combined and dried. The four purified metabolites possess the following physico-chemical properties.

Miramide A (**1**): Pale yellow oil; 0.6 mg; UV λ_max_ (MeOH) 220 nm. ^1^H and ^13^C NMR data, see Table S2; HR-ESI-MS *m/z* 592.3239 [M + H]^+^ (calc. 592.3234; 591.3161 [M]^+^; molecular Formula C_30_H_45_N_3_O_9_).

Miramide B (**2**) and C (**3**): Mixture; pale yellow oil; 0.8 mg; UV λ_max_ (MeOH) 220 nm. ^1^H NMR data, see Table S3; HR-ESI-MS *m/z* 606.3384 [M + H]^+^ (calc. 606.3390; 605.3306 [M]^+^; molecular Formula C_31_H_47_N_3_O_9_).

Miramide D (**4**): Yellow oil; 0.3 mg; UV λ_max_ (MeOH) 220 nm. ^1^H NMR data, see Table S4; HR-ESI-MS *m/z* 578.3068 [M + H]^+^ (calc. 578.3077; 577.2990 [M]^+^; molecular Formula C_29_H_43_N_3_O_9_).

### 2.6. Nuclear Magnetic Resonance (NMR) Spectroscopy

1D and 2D NMR spectra were acquired in deuterated water (Deutero, Kastellaun, Germany) on a Bruker Avance Neo 500 spectrometer equipped with a 5 mm TXI prodigy cryoprobe (Bruker BioSpin GmbH, Rheinstetten, Germany) at 298 K. NMR chemical shifts were reported in parts per million (ppm) relative to the residual solvent signal D_2_O at δ 4.79. NMR data were analyzed using Topspin, version 4.06 (Bruker, Biospin GmbH, Rheinstetten, Germany) and ACD/Spectrus Processor 2020.2.1 (ACD/Labs, Toronto, Canada).

## 3. Results and Discussion

### 3.1. Identification and Heterologous Expression of the Hybrid NRPS-PKS Gene Cluster from S. mirabilis Lu17588 into S. albus Del14

The analysis of the genome sequence of *Streptomyces mirabilis* Lu17588 with antiSMASH software [26] led to the identification of a putative NRPS-PKS cluster for which no similar known BGC was shown and thus might be interesting for further examination. To characterize the identified NRPS-PKS gene cluster, we set out to express the cluster heterologously. Analysis of the previously constructed and sequenced genomic library of the strain did not reveal any cosmid covering the entire gene cluster. To reconstruct the cluster, three overlapping cosmids 11_E04, 06_C04 and 08_G05 were selected from the library.

**Figure 1:**
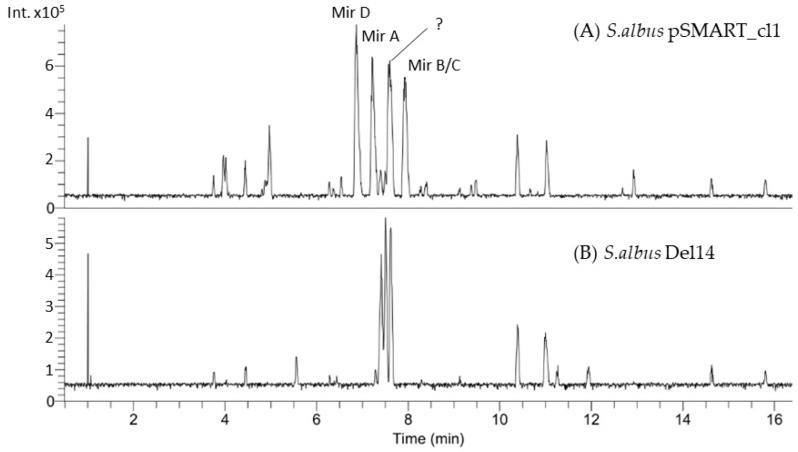
Section of the LC-MS chromatogram showing the mutant strain *S. albus* pSMART_cl1, which was found to produce four new metabolites with *m/z* values of 592.3, 606.3 and 578.3 (**A**). The host strain *S. albus* Del14 lacking the construct was used as a control (**B**).

The chromosomal fragments were cloned into the cosmids, which were cut out by restriction digestion and assembled into the BAC vector pSMART_assmbl using TAR recombination in Saccharomyces cerevisiae [21,22]. The constructed BAC pSMART_cl1 contains a 90 kb chromosomal fragment of *S. mirabilis* with the entire NRPS-PKS gene cluster (GenBank accession number: OM108703). The BAC pSMART_cl1 was transferred into the heterologous host strain Streptomyces albus Del14 by conjugation [16] (Table S1). The obtained exconjugant strain *S. albus* pSMART_cl1 and the control strain without the BAC S. albus Del14 were fermented in the production medium DNPM in triplicate to ensure stable production. Secondary metabolites were extracted from the culture broth of S. albus pSMART_cl1 and S. albus Del14 with n-butanol, and the extracts were analyzed by HPLC-MS. This analysis led to the identification of several new peaks at RT 6.8, 7.2, 7.5 and 7.9 with m/z values of 577.2, 591.3, 591.3 and 605.3, respectively, in the extract of S. albus pSMART_cl1 (Figure 1). The identified peaks were not present in the extract of the control strain S. albus Del14, indicating that the peaks originate from cluster expression (Figure 1). The extract of S. albus pSMART_cl1 was additionally analyzed using high-resolution HPLC-MS, and the calculated molecular weights of the identified compounds (605.3306 Da, 591.3161 Da and 577.2990 Da) were used for the search in natural product databases. This survey did not reveal any known natural products with the identified molecular masses, implying that the detected compounds might be new. To obtain structural information about the identified natural products, purification and structure elucidation were performed.

### 3.2. Purification and Structure Elucidation of the New Metabolites

**Figure 2:**
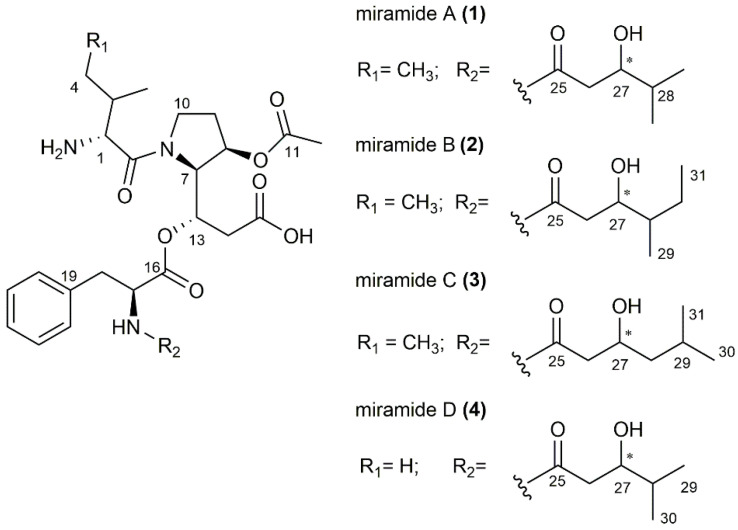
Chemical structures of miramides A–D (**1**–**4**). The core structure is composed of Ile or Val in the case of miramide D, Det and Phe. The non-amino acid side chain varies for most derivatives; only miramides A and D bear the same 3-hydroxy-4-methylpentanoyl moiety. The stereocenter in the fatty acid chain is marked with an asterisk.

The four new compounds miramides A–D, which were produced from *S. albus* pSMART_cl1 bearing the hybrid NRPS-PKS gene cluster, were isolated from a 10 L culture using the production medium DNPM. The supernatant was extracted with butanol, and the targeted metabolites were purified over several chromatographic steps. Three of the four newly identified compounds (Mir D-RT 6.8; Mir A–RT 7.2; Mir B/C–RT 7.9), as shown in the chromatogram (Figure 1), were obtained in sufficient amounts and purity. The structures of miramides A–D with *m/z* 591.318 (A, 0.6 mg; RT 7.2), 605.329 (B and C, 0.8 mg; RT 7.9) and 577.298 (D, 0.3 mg; RT 6.8) (Figures S2–S4) were elucidated by analysis of 1D and 2D NMR spectroscopic data (Figure 1 and Figure 2).

Miramide A (**1**) was obtained as a pale-yellow oil with UV absorption at 220 nm. The molecular formula was determined as C_30_H_45_N_3_O_9_ from the molecular ion peak at *m/z* 592.3239 ([M + H]^+^ (591.3161 [M], calc. 592.3234) (Figure S2). ^1^H and HSQC spectra showed the presence of 5 methyl, 6 methylene and 13 methine groups (Figures S5 and S6). The remaining 6 quaternary carbons were assigned from HMBC spectra (Figure S7). A number of related proton/carbon chemical shifts characteristic of amino acids led us to assume that the structure has at least a partially peptide nature. In-depth analysis of HMBC, COSY and selective 1D TOCSY spectra revealed the amino acid sequence Ile-Det-Phe (Figures S6–S10). This is supported by key COSY cross peaks between Ile protons H-1/H-2, H-2/H-3 and H-4/H-5 and HMBC correlations from Det H-7 to Ile carboxyl C-6 establishing the amide bond between the first two amino acids. The unusual γ amino acid Det is based on proline, bearing two modifications. First, a hydroxyl group is attached in the β position; second, the carboxyl group of proline is extended with a malonate unit, and subsequently, the keto group is reduced to an alcohol and thus available for a depsipeptide bond to the next amino acid. The COSY correlations H-7/H-8, H-7/H-13, H-13/H-14 and key HMBC signals from H-7 to C-8, C-9, C-13 and H-13 to C-7, C-14 and C-15 were observed, confirming the presence of Det. Additionally, an acetyl group (δ_C_ 20.2/δ_H_ 2.05, δ_C_ 173.1) was found to be attached to the hydroxyl at C-8, which is supported by HMBC signals from Det H-8 to acetyl C-11. Further detection of aromatic methine protons H-20–H-24 and COSY cross peaks from methylene protons H-18 (δ_H_ 3.15 and 2.92) to CH_α_-17 (δ_H_ 4.64) supported the presence of the last amino acid Phe. The HMBC signal from Det H-13 to Phe C-16 (δ_C_ 171.3) revealed the connection between Phe and Det via the hydroxyl group at C-13 forming a depsipeptide. Finally, a short fatty acid chain 3-hydroxy-4-methylpentanoyl bound to the N-terminus of Phe was found. The COSY correlations were detected between H-26/H-27, H-27/H-28 and H-28/H-29/H-30. To further support these findings, selective 1D TOSCY experiments were performed to distinguish the methyl groups within this part from those in Ile. For instance, irradiation of methyl H-3 at δ_H_ 1.06 ppm revealed a triplet at δ_H_ 0.90, indicating the methyl group H-5 in Ile (Table S2, Figure S9). Irradiation at 0.76 ppm (H-29/H-30) produced two clear signals for methine protons H-27 (δ_H_ 3.56) and H-28 (δ_H_ 1.47), supporting the presence of the 3-hydroxy-4-methylpentanoyl moiety (Figure S10).

Miramide B (**2**) and miramide C (**3**) were obtained as a mixture that was yellow coloured, had an oily consistency and exhibited UV absorption at 220 nm. The molecular formulas were both established as C_31_H_47_N_3_O_9_ from the exact mass (Figure S3). The ^1^H and HSQC spectra were close to those of miramide A and showed that both compounds correspond to the amino acid sequence Ile-Det-Phe (Table S3, Figure S11). However, the mass difference of 14 Da between miramide A and B/C indicated an additional methyl group. However, signal overlaps, especially in the area of the methyl groups (0–1.5 ppm; Figure S11), made it difficult to clearly interpret the proton spectrum. Therefore, selective 1D TOCSY spectra were performed to separate the Ile methyl groups from those of the fatty acid residue and to analyze the two different side chains in miramides B and C. Excitation of methyl group H-5 (δ_H_ 0.88), for instance, causes resonances for methine H-1 and H-2, methyl group H-3 and methylene H-4, indicating the affiliation of this methyl to Ile (Figure S12). Selective excitation of H-27 (δ_H_ 3.79) in B resulted in the appearance of resonances for methine H-28, methylenes H-30 and the two methyl groups H-29 (doublet) and H-31 (triplet), suggesting a 3-hydroxy-4-methylhexanoyl moiety instead of a 3-hydroxy-4-methyl-pentanoyl moiety, as found in miramide A (Figure S13). Similarly, 1D TOCSY excitation of proton H-27 (δ_H_ 3.91) in C produces signals for methylenes H-28, methine H-29 and two isopropyl methyl groups H-30 and H-31, confirming a 3-hydroxy-5-methylhexanoyl moiety (Figure S14).

Detailed analysis of the 1D and 2D NMR spectra of miramide D (**4**), C_29_H_43_N_3_O_9,_ revealed almost identical signals as those found for miramide A (Table S4, Figure S15). Only resonances for methyl H-5 in the Ile moiety were missing. Instead, two doublets of an isopropyl group appeared (H-3 and H-4), indicating a valine was present instead of an isoleucine. This is particularly evident in the selective 1D TOCSY experiment when methyl H-3 at δ_H_ 1.03 is excited and a second doublet appeared at δ_H_ 0.94 (Figure S16). Furthermore, excitation at δ_H_ 3.61 (H-27) exposed two doublet methyls at δ_H_ 0.77 and 0.78 (H-29 and H-30) and a methine at δ_H_ 1.51 (H-28), verifying the same 3-hydroxy-4-methylpentanoyl moiety as that found in miramide A (Figure S17).

The absolute stereochemistry of isolated miramides was not determined. However, the stereochemistry of all amino acid residues found in the structures of rimosamides and detoxins, which are structurally very related to miridamides, was assigned to the L configuration [17]. According to this, we assume that amino acid residues of miramides also have an L configuration. To elucidate the configuration of the stereocentre in the 3-hydroxy-4-methyl pentanoyl moiety, Mosher’s method was performed for miramide A [27,28]. However, this did not lead to applicable results due to probable degradation during the reaction process. The bioactivity of the isolated compounds has not been tested due to very low amounts of pure compound for each derivative.

**Figure 3:**
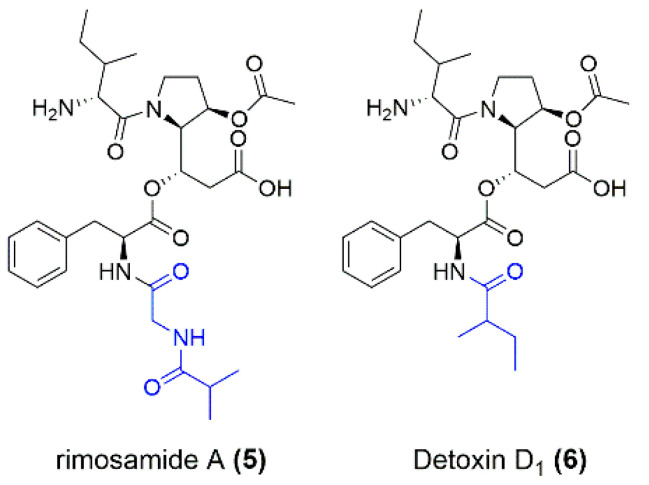
Structures of rimosamide A (**5**) and detoxin D_1_ (**6**) as examples of structural differences from the isolated compounds miramides A–D. The moiety (marked in blue) attached to Phe differs for rimosamides, detoxins and miramides.

In summary, four new depsipetide compounds (miramides A–D) that contained a new hydroxy fatty acid moiety attached to Phe were identified. A database search with parts of the miramide structure revealed that similar compounds exist, namely, rimosamides A–D [18] and detoxins D1–D5 [17]. Compared to miramides, rimosamides have a Gly-Ibu moiety instead of the hydroxy fatty acid sidechain [18] and detoxins have variations in fatty acid residues, which are attached via amide bonds to Phe [17] (Figure 3).

### 3.3. Biosynthetic Gene Cluster and Derivation of Biosynthesis of Miramides A–D

**Figure 4:**
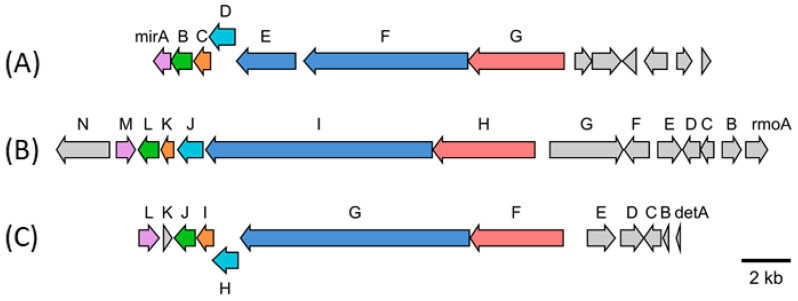
BGC of miramides (**A**) from *S. mirabilis* Lu17588, rimosamides (**B**) from *S. rimosus* NRRL B-2659 and detoxins (**C**) from *S. caespitosus* var. *detoxicus* 7072. Homologous genes are displayed with the same colors. The NRPS genes responsible for the construction of the core part Ile (Val)-Det-Phe are shown in red and blue. The following other similar genes within the three clusters are shown: SpcZ (purple), taurine dioxygenase (green), putative protein (orange) and GSCFA family protein (light blue).

Expression of a 90 kb chromosomal fragment of *S. mirabilis* cloned in the BAC pSMART_cl1 led to the production of various miramides in *S. albus* Del14. The profound structural similarity of the isolated compounds to rimosamides and detoxines implied similarities in the encoding BGC [17,18]. Direct comparison of the protein sequences of the enzymes encoded by the genes within the expressed chromosomal fragment of *S. mirabilis* with the protein sequences of the enzymes involved in rimosamide and detoxin biosynthesis allowed us to narrow down the putative miramide gene cluster to seven genes: *mirA*–*mirG* (Figure 4, Table 1). The genes *mirE*, *mirF* and *mirG* encode putative NRPS-PKS enzymes responsible for the assembly of the depsipeptide backbone of miramides. The gene *mirB*, which encodes a putative dioxygenase, is probably involved in the tailoring steps of miramide biosynthesis. The genes *mirA*, *mirC* and *mirD* encode hypothetical proteins without clear function in miramide biosynthesis.

**Figure 5:**
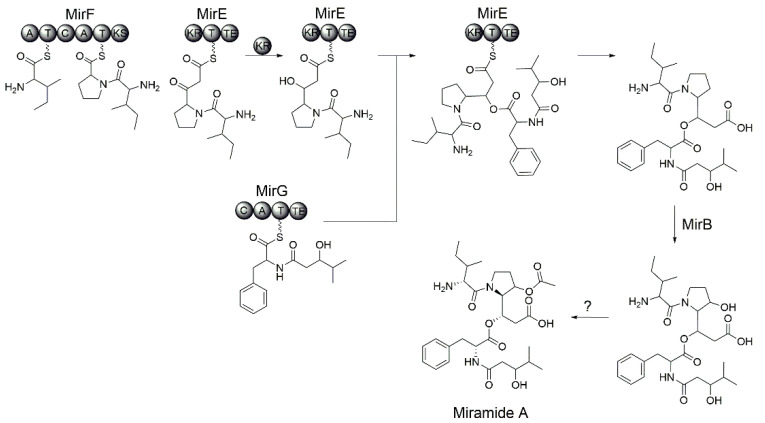
Proposed biosynthetic scheme of miramide A.

The biosynthetic scheme for miramides was proposed based on the rimosamide biosynthetic pathway. The genes *mirF* and *mirE* encode a hybrid NRPS-PKS assembly line. The MirF and MirE proteins have the following domain organization: A-T-C-A-T-KS and KR-T-TE, respectively. Functionally, the proteins MirF and MirE correspond to the single NRPS-PKS protein RmoI that was involved in the biosynthesis of rimosamides (Table 1). Similar to RmoI, the adenylation domains of MirF exhibit specificity to isoleucine and proline. Therefore, we propose that MirF builds the Ile-Pro dipeptidyl intermediate (Val-Pro in the case of miramide D). The formed precursor is further elongated with malonate (Figure 5). Since MirF and MirE lack an acyltransferase domain, we propose that an unidentified standalone acyltransferase loads in trans the malonate residue onto the ACP domain of MirE. The C-terminal KS-domain of MirF catalyzes the Claisen condensation between the dipeptidyl intermediate (Ile-Pro or Val-Pro) and malonate. The ketoreductase domain of MirE reduces the ketone to an alcohol. The formed intermediate, Val/Ile-Det, is involved in depsipeptide bond formation with the Phe-fatty acid precursor provided by MirG (Figure 5).

The MirG NRPS protein shows homology to the rimosamide biosynthetic enzyme RmoH (Table 1) and shares with it the following domain organization: C-A-T-TE. The starter condensation domain, which was found in the case of MirG, was reported to be involved in the incorporation of fatty acids. The amino acid specificity of the adenylation domain of MirG cannot be predicted. We propose that MirG catalyzes the condensation of several hydroxylated fatty acids provided by host metabolism with the phenylalanine residue. The formed fatty acid-Phe intermediate is transferred to the thioesterase domain of MirG and subsequently reacts with the MirE-bound precursor to form a depsipeptide bond (Figure 5).

To build miramides A–D, the proline residue of the nascent depsipeptide must be hydroxylated and then acetylated. Similar to rimosamide biosynthesis, we propose that the putative taurine dioxygenase MirB, which is homologous to RmoL, is involved in the hydroxylation step. Since no dedicated acetyltransferase could be identified in the miramide or rimosamide gene clusters, it is not clear which enzyme catalyzes the acetylation step. It cannot be excluded that one of the hypothetical genes (*mirA*, *mirC* or *mirD*) encoded within the miramide gene cluster is responsible for this biosynthetic step.

## 4. Conclusions

In this paper, we describe the successful heterologous expression of a hybrid NRPS-PKS gene cluster from *S. mirabilis* Lu17588 in *S. albus* Del14. LC-MS analysis of the resulting strain led to the identification of four new depsipeptides–miramides A–D. The new compounds were purified, and the chemical structures were elucidated by NMR spectroscopy. The newly identified depsipeptides differ from the structurally similar rimosamides and detoxins as various unique short hydroxylated fatty acid residues are present in the miramide structures. Based on the BGC and literature analyses, a biosynthetic pathway leading to the production of miramides A–D was proposed.

## Figures and Tables

**Table 1:** Genes and putative gene functions in the miramide BGC and their respective homologs in the rimosamide (*Rmo*) and detoxin (*Det*) gene clusters.

Gene; Locus Tag	Putative Product (Homologue Accession Number)	Homologue in Rmo/Det Pathway
*mirA*; LU17588_063290	SpcZ, hypothetical protein (WP_004571773.1)	*rmoM*, *detL*
*mirB*; LU17588_063300	Alpha-ketoglutarate-dependent taurine dioxygenase (WP_004571774.1)	*rmoL*, *detJ*
*mirC*; LU17588_063310	Putative protein (WP_032920603.1)	*rmoK*, *detI*
*mirD*; LU17588_063320	GSCFA family protein (WP_030179607.1)	*rmoJ*, *detH*
*mirE*; LU17588_063330	Polyketide synthase; KR-T-TE (WP_004571777.1)	*rmoI*, *detG*
*mirF*; LU17588_063340	Hybrid nonribosomal peptide synthetase-polyketide synthase; A-T-C-A-T-KS (WP_004571777.1)	*rmoI*, *detG*
*mirG*; LU17588_063350	Nonribosomal peptide synthetase C-A-T-TE (WP_078586793.1)	*rmoH*, *detF*

## Data Availability

Not applicable.

## Supplementary Materials

The following information is available online at https://www.mdpi.com/article/10.3390/microorganisms10091752/s1. Table S1: Bacterial strains, cosmids and BACs used in this study. Table S2: NMR spectroscopic data of miramide A (**1**) in D_2_O (500 MHz). Table S2: NMR spectroscopic data of miramide B_1_ (**2**) and B_2_ (**3**) in D_2_O (500 MHz). Table S3: NMR spectroscopic data of miramide C (**4**) in D_2_O (500 MHz). Table S4: Genes and putative gene functions in miramide BGC according to antiSMASH and MiBIG results. Figure S1: Assembly of the *S. mirabilis* chromosomal fragment containing the miramide biosynthetic gene cluster. A–Schematic representation of the chromosomal fragment with the miramide gene cluster and of the cosmids that cover the fragment. B–TAR assembly of the miramide biosynthetic gene cluster on the pSMART_assembl backbone. The homology arms used for assembly are shown as rectangles with the letters L and R. Figure S2: HRMS spectra of miramide A (**1**). Figure S3: HRMS spectra of miramide B (**2**) and C (**3**). Figure S4: HRMS spectra of miramide D (**4**). Figure S5: 1H NMR spectrum of miramide A (**1**) measured in D_2_O (500 MHz). Figure S6: HSQC spectrum of miramide A (1) measured in D_2_O (500 MHz). Figure S7: HMBC spectrum of miramide A (**1**) measured in D_2_O (500 MHz). Figure S8: ^1^H,^1^H-COSY spectrum of miramide A (**1**) measured in D_2_O (500 MHz). Figure S9: 1D selective TOCSY spectrum of miramide A (**1**) measured in D_2_O (500 MHz), irradiation at 1.06 ppm (H-3). Figure S10: 1D selective TOCSY spectrum of miramide A (**1**) measured in _D2O_ (500 MHz), irradiation at 0.76 ppm (H-29/H-30). Figure S11: 1H NMR spectrum of miramide B (**2**)/miramide C (**3**) measured in D_2_O (500 MHz). Figure S12: 1D selective TOCSY spectrum of miramide B (**2**)/miramide C (**3**) measured in D_2_O (500 MHz), irradiation at 0.88 ppm (H-2). Figure S13: 1D selective TOCSY spectrum of miramide B (**2**)/miramide C (**3**) measured in D_2_O (500 MHz), irradiation at 3.79 ppm (H-27, B1). Figure S14: 1D selective TOCSY spectrum of miramide B (**2**)/miramide C (**3**) measured in D_2_O (500 MHz), irradiation at 3.91 ppm (H-27, B_2_). Figure S15: 1H NMR spectrum of miramide D (4) measured in D_2_O (500 MHz). Figure S16: 1D selective TOCSY spectrum of miramide D (**4**) measured in D_2_O (500 MHz), irradiation at 1.03 ppm (H-3). Figure S17: 1D selective TOCSY spectrum of miramide D (**4**) measured in D_2_O (500 MHz), irradiation at 3.61 ppm (H-27). References in Supplementary Materials can been seen [16,29].
