# Supplementary material for: Miramides A–D: Identification of Detoxin-like Depsipeptides after Heterologous Expression of a Hybrid NRPS-PKS Gene Cluster from Streptomyces mirabilis Lu17588
Source: Microorganisms. 2022 Aug 30;10(9):1752. doi: 10.3390/microorganisms10091752 (PMC9503745; doi:10.3390/microorganisms10091752)
Supplement: Supplementary file 1 [file microorganisms-10-01752-s001.zip › microorganisms-1821711-supplementary.pdf]

Supporting Information

Miramides A-D, detoxin-like depsipeptides identified after heterologous Expression of a hybrid NRPS-PKS gene cluster from *Streptomyces mirabilis* Lu17588

Table S1. Bacterial strains, cosmids and BACs used in this study.

Bacterial strain	Function	Reference
Streptomyces mirabilis Lu17588	Strain harboring miramide gene cluster	This work.
Streptomyces albus pSMART_cl1	*S. albus* Del14 strain harboring BAC pSMART_cl1	This work.
Streptomyces albus Del14	Heterologous expression host	[1]
E. coli GB05 redCC	Strain for cosmid assembly and cloning	
E. coli ET12567 pUB 307	Strain for cosmid assembly and cloning	[2]
**BACs**		
pSMART_assembl	AmR; BAC vector	Lucigen (USA)
pSMART_cl1	BAC vector containing chromosomal fragment of *S. mirabilis*	This work.
**Cosmid**		
Cos15A_gus	Cloning vector	This work.

Table S2. NMR spectroscopic data of miramide A (**1**) in D_2O (500 MHz).

position	δ_C[a]	δ_H[b]	$^1H,^1H$-COSY	HMBC	1D select. TOCSY
1	56.1, CH	4.20 (d, 4.6)	2	2, 3, 4, 6	
2	35.8, CH	1.97 (m)	1, 3	-	
3	14.5, CH_3	1.06 (3H.d, 7.0)	2	1, 2, 4	1, 2, 4, 5
4	23.2, CH_2	1.47 (m)<br>1.16 (m)	2, 5	-	
5	10.6, CH_3	0.90 (t, 7.3)	4	2, 4	
6	169.7, C	-	-	-	
7	58.6, CH	4.71 (m)**	-	-	
8	71.4, CH	5.16 (q, 7.3)	7, 9	10, 11, 13	
9	29.5, CH_2	2.21*<br>1.80 (q, 2x 6.5)	10	8	
10	45.7, CH_2	3.70 (m)	9	8	

		3.56 (m)			
11	173.1, C	-	-	-	
12	20.2, CH_3	2.05 (3H, s)	-	11	
13	71.8, CH	5.59 (td, 7.0, 3.0)	7, 14	8, 15, 16	
14	38.9, CH_2	2.43 (m)<br>2.38 (m)	13<br>13	7, 13, 15<br>7, 13, 15	
15	177.2, C	-	-	-	
16	171.3, C	-	-	-	
17	54.1. CH	4.64 (m)**	-	-	
18	36.1, CH_2	3.15 (dd, 14.0, 6.4)<br>2.92 (dd, 14.0, 9.0)	17	16, 17, 19, 20, 24	
19	136.3, C	-	-	-	
20,24	128.9, CH	7.22 (m)	21, 23	18, 22	
21,23	128.6, CH	7.30 (m)	20, 24	19, 20, 24	
22	126.9, CH	7.23 (m)	21, 23	20, 24	
25	174.0, C	-	-	-	
26	40.0, CH_2	2.29 (dd, 14.4, 4.2)<br>2.20 (dd, 14.4, 9.0)	27	25, 27, 28	
27	73.1, CH	3.56 (m)*	26	25, 28, 29, 30	
28	32.6, CH	1.47 (m)*	29, 30	27, 29, 30	
29	17.7, CH_3	0.76 (3H, d, 7.0)	28	27, 28, 30	27, 28
30	16.3, CH_3	0.76 (3H, d, 7.0)	28	27, 28, 29	

*overlap

** under water signal

[a]followed by multiplicity, ^{13}C chemical shifts were all taken from HSQC and HMBC spectra

[b]followed by multiplicity and coupling constants *J* in Hz

Table S3. NMR spectroscopic data of miramide B (**2**) and C (**3**) in D_2O (500 MHz).

	**Miramide B**	**Miramide C**	
position	δ_H[a]	δ_H[a]	1D select. TOCSY
1	4.18[b] (d, 4.5)	4.16[b] (d, 4.5)	
2	2.01 (m)	2.02 (m)	
3	1.04[b] (3H, d, 7.0)	1.03[b] (3H, d, 7.0)	
4	1.47 (m)<br>1.14 (m)	1.47 (m)<br>1.14 (m)	
5	0.88 (m)	0.88 (m)	1, 2, 3, 4
6	-	-	
7	n.d. **	n.d. **	
8	5.21 (q, 7.7)	5.21 (q, 7.7)	
9	n.d.*	n.d.*	
10	3.68 (m)<br>3.64 (m)	3.68 (m)<br>3.64 (m)	
11	-	-	
12	2.09 (s)	2.09 (s)	
13	5.51 (m)	5.51 (m)	
14	n.d. *	n.d. *	

15	-	-	
16	-	-	
17	n.d. **	n.d. **	
18	3.14 (dd, 5.7, 14.0)<br>2.98 (dd, 8.5, 14.0)	3.14 (dd, 5.7, 14.0)<br>2.98 (dd, 8.5, 14.0)	
19	-	-	
20, 24	7.20 (m)	7.20 (m)	
21, 23	7.30 (m)	7.30 (m)	
22	7.25 (m)	7.25 (m)	
25	-	-	
26	2.30 (dd, 14.5, 5.0)<br>2.34 (dd, 14.5, 5.0)	2.29 (2H, d, 6.8)	
27	3.79 (m)	3.91 (m)	26, 28, 29, 30, 31
28	1.27 (m)	1.30 (m)<br>1.12 (m)	
29	1.30 (m)<br>1.06 (m)	1.58 (1H, m)	
30	0.78 (3H, t, 7.0)	0.80 (3H, d, 7.0)	
31	0.76 (3H, d, 7.0)	0.77 (3H, d, 7.0)	

*overlap

** under water signal

[a]followed by multiplicity and coupling constant *J* in Hz

[b]can be changed within a line

Table S4. NMR spectroscopic data of miramide D (**4**) in D_2O (500 MHz).

position	δ_H[a]	1D select. TOCSY
1	4.08 (m)	
2	2.21 (m)	
3	1.03 (d, 6.9)	1, 2, 4
4	0.94 (d, 6.9)	
5		
6	-	
7	n.d. **	
8	5.19 (q, 7.7)	
9	n.d. *	
10	3.67 (m)<br>3.62 (m)	
11	-	
12	2.09 (s)	
13	5.50 (m)	
14	n.d. *	
15	-	
16	-	
17	n.d. **	
18	3.14 (dd, 5.7, 14.1)<br>2.98 (dd, 8.5, 14.1)	

19	-	
20, 24	7.20 (m)	
21, 23	7.30 (m)	
22	7.25 (m)	
25	-	
26	2.37 (dd, 14.5, 4.0)<br>2.23 (dd, 14.5, 9.0)	
27	3.61 (m)	26, 28, 29, 30
28	1.51 (m)	
29	0.77 (d, 6.9)	
30	0.78 (d, 6.9)	

*overlap

** under water signal

[a]followed by multiplicity and coupling constant *J* in Hz

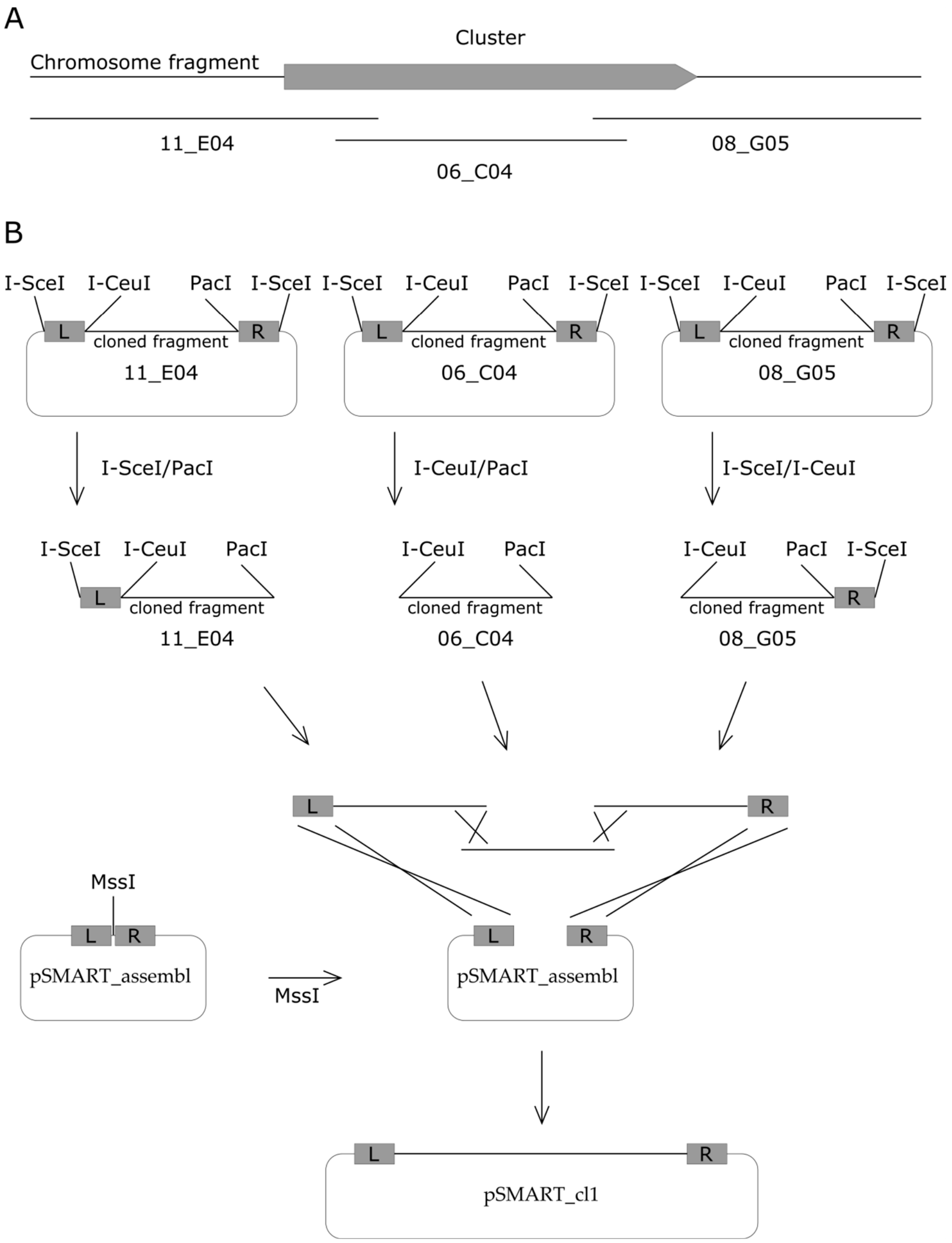

Figure S1. Assembly of the *S. mirabilis* chromosomal fragment containing the miramide biosynthetic gene cluster. A – Schematic representation of the chromosomal fragment with the miramide gene cluster and of the cosmids that cover the fragment. B – TAR assembly of the miramide biosynthetic gene cluster on the pSMART_assembl backbone. The homology arms used for assembly are shown as rectangles with the letters L and R.

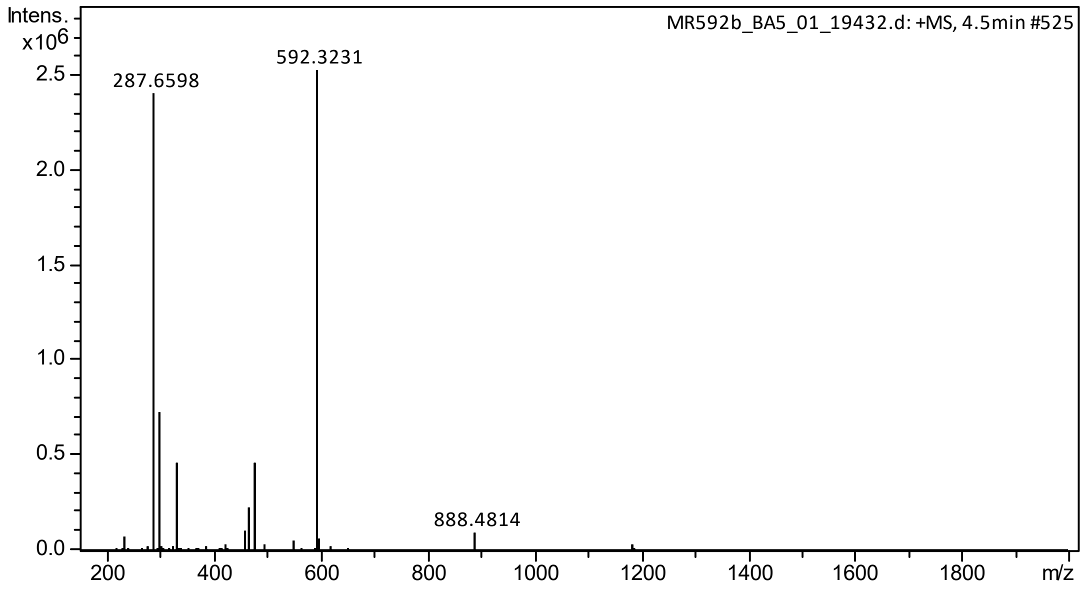

Figure S2. HRMS spectra of miramide A (**1**).

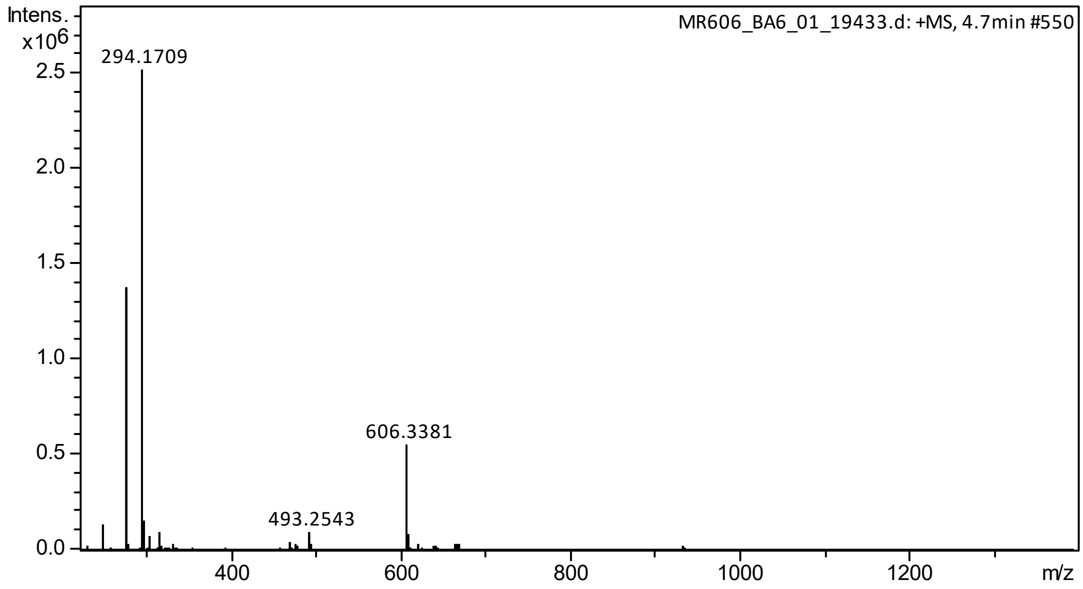

Figure S3. HRMS spectra of miramide B (**2**) and C (**3**).

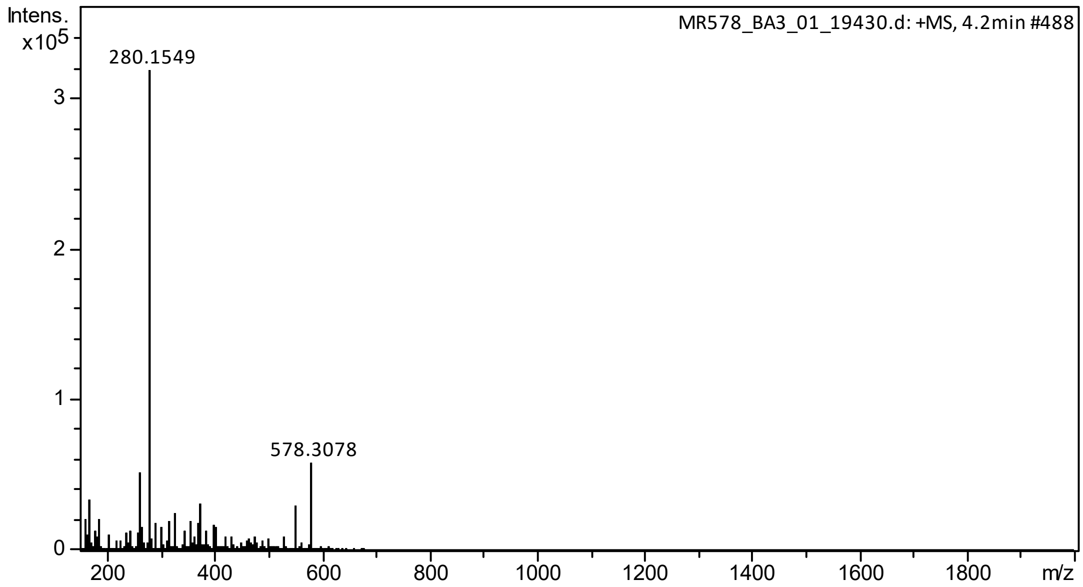

Figure S4. HRMS spectra of miramide D (**4**).

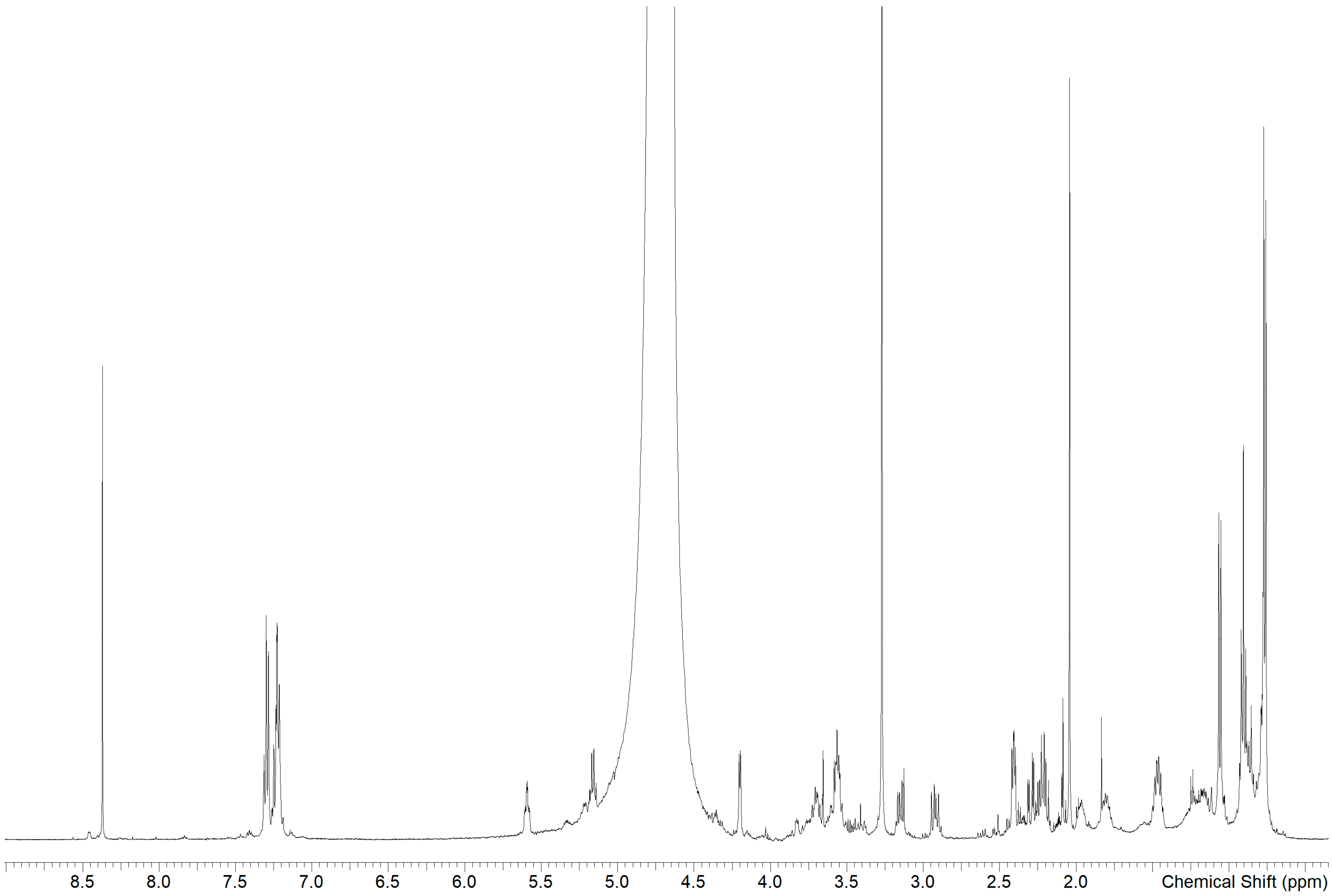

Figure S5. ^{1}H NMR spectrum of miramide A (**1**) measured in D_2O (500 MHz).

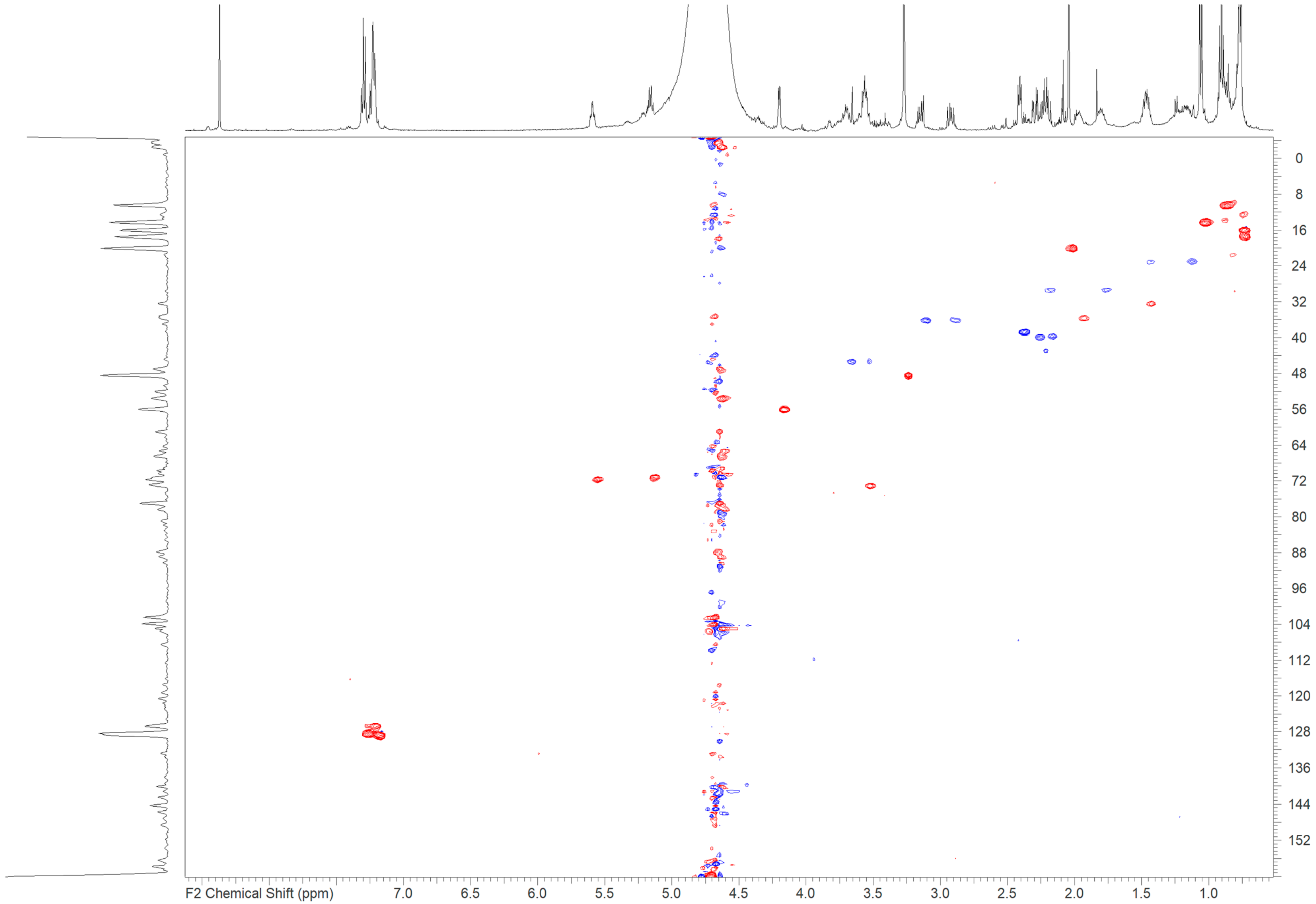

Figure S6. HSQC spectrum of miramide A (**1**) measured in D_2O (500 MHz).

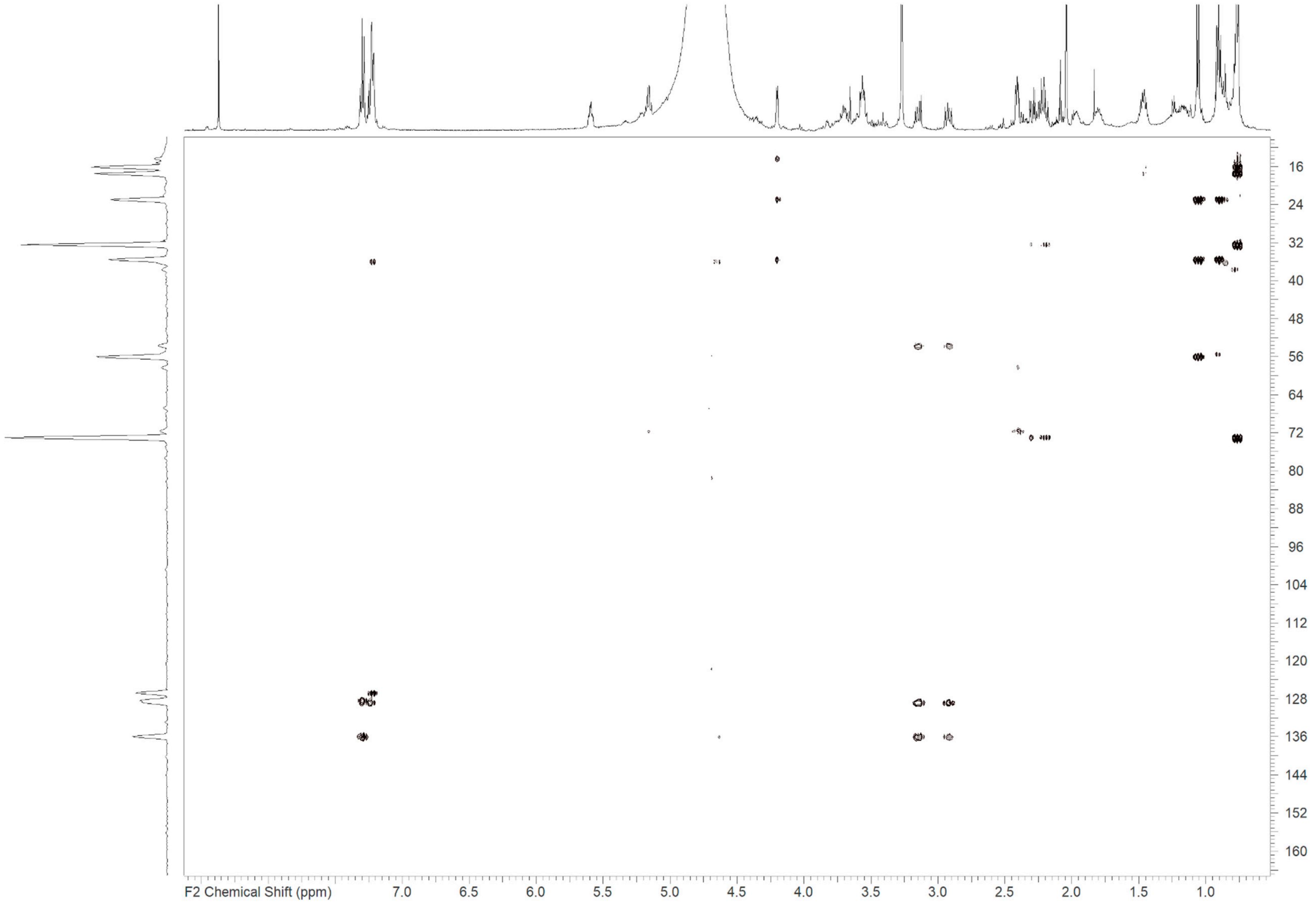

Figure S7. HMBC spectrum of miramide A (**1**) measured in D_2O (500 MHz).

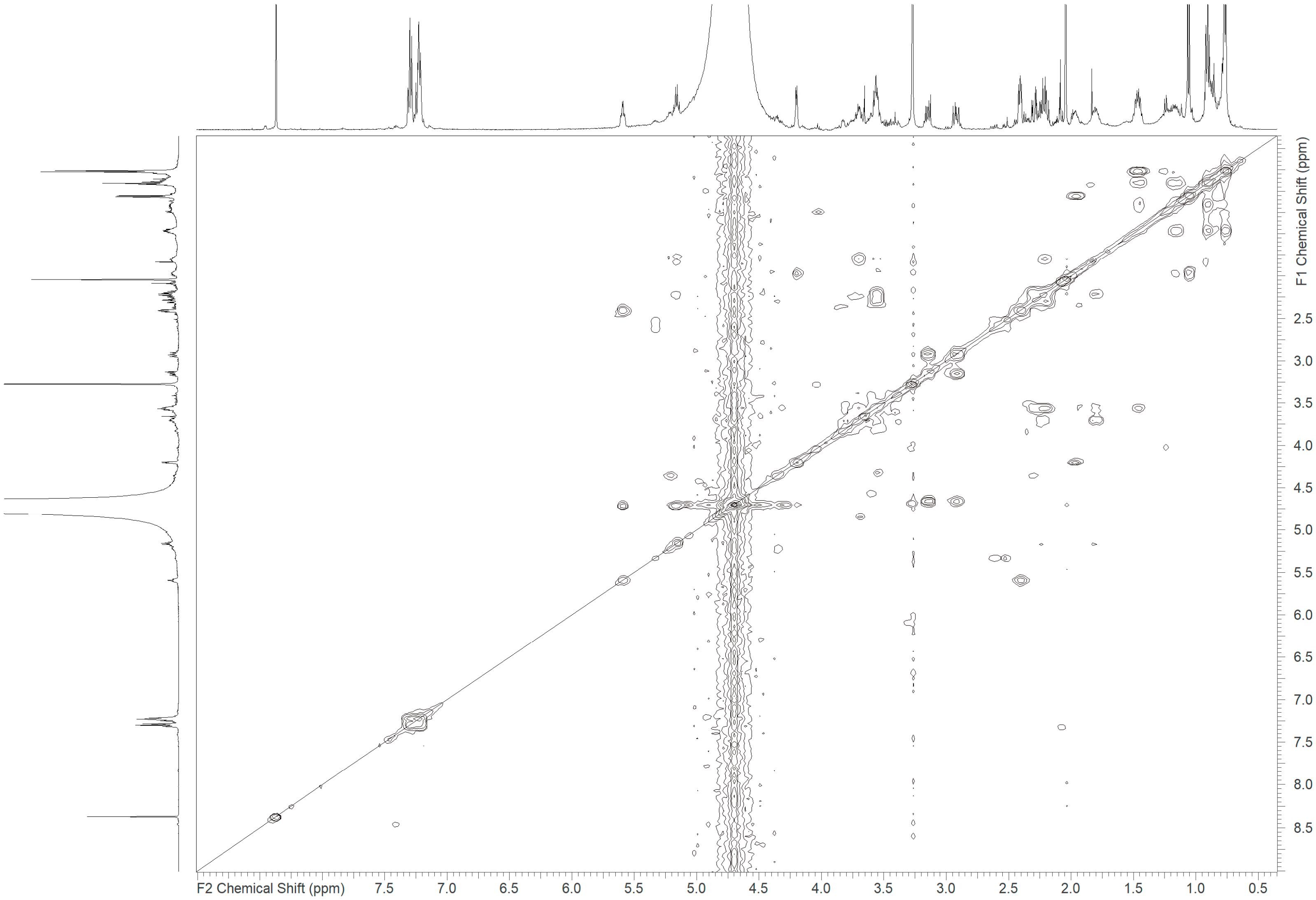

Figure S8. ^{1}H,^{1}H-COSY spectrum of miramide A (**1**) measured in D_2O (500 MHz).

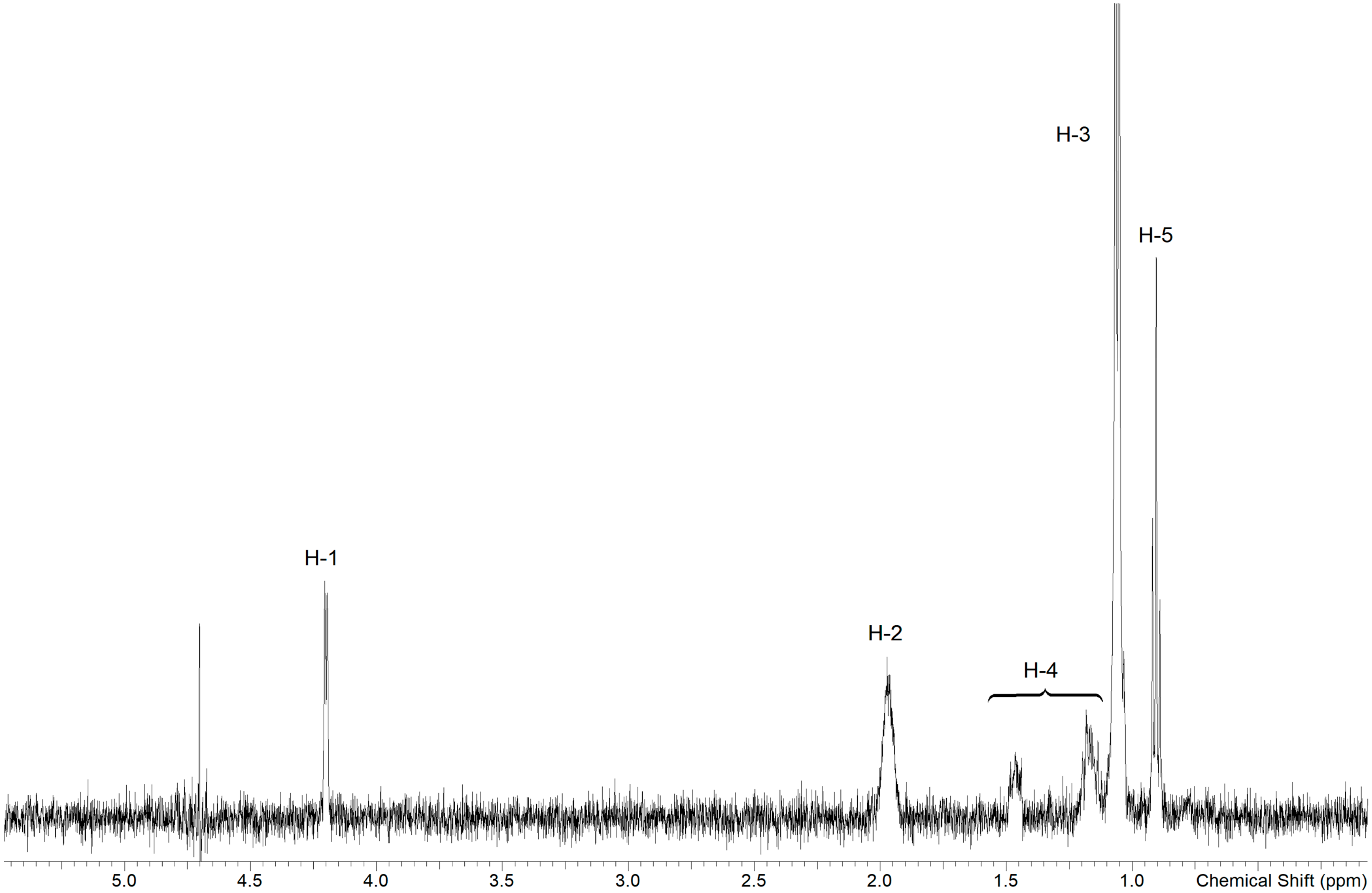

Figure S9. 1D selective TOCSY spectrum of miramide A (**1**) measured in D_2O (500 MHz), irradiation at 1.06 ppm (H-3).

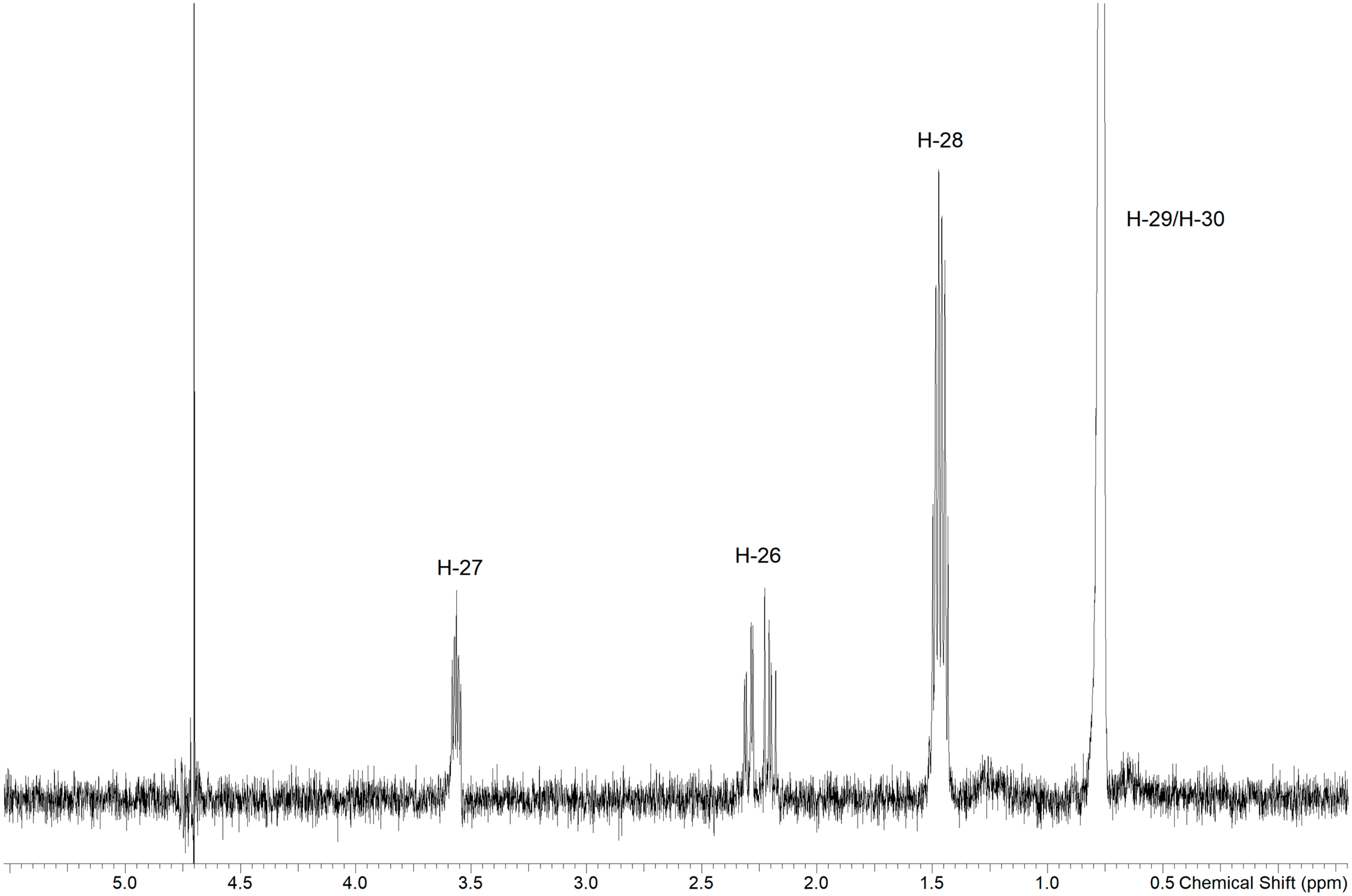

Figure S10. 1D selective TOCSY spectrum of miramide A (**1**) measured in D_2O (500 MHz), irradiation at 0.76 ppm (H-29/H-30).

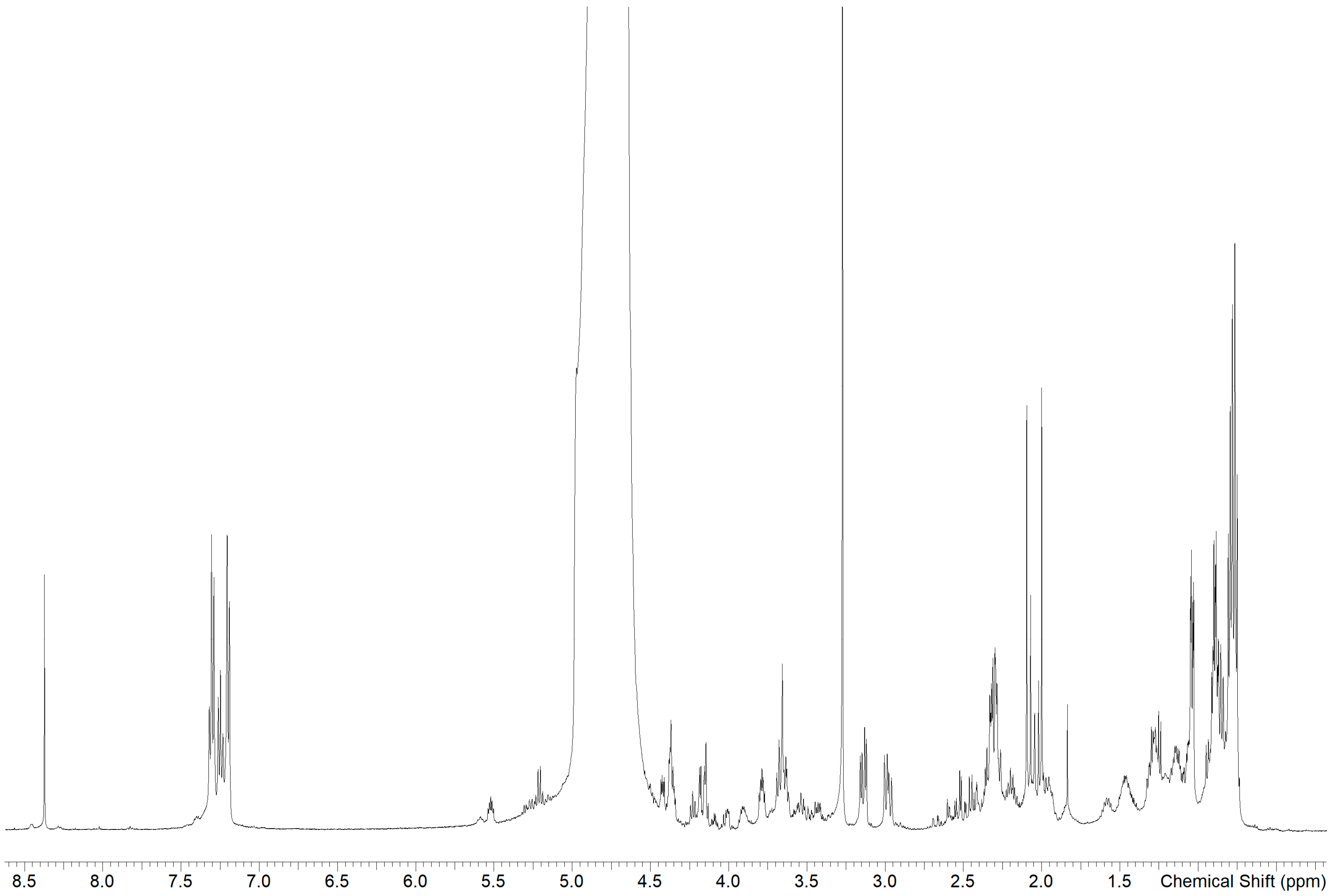

Figure S11. ^{1}H NMR spectrum of miramide B (**2**)/ miramide C (**3**) measured in D_2O (500 MHz).

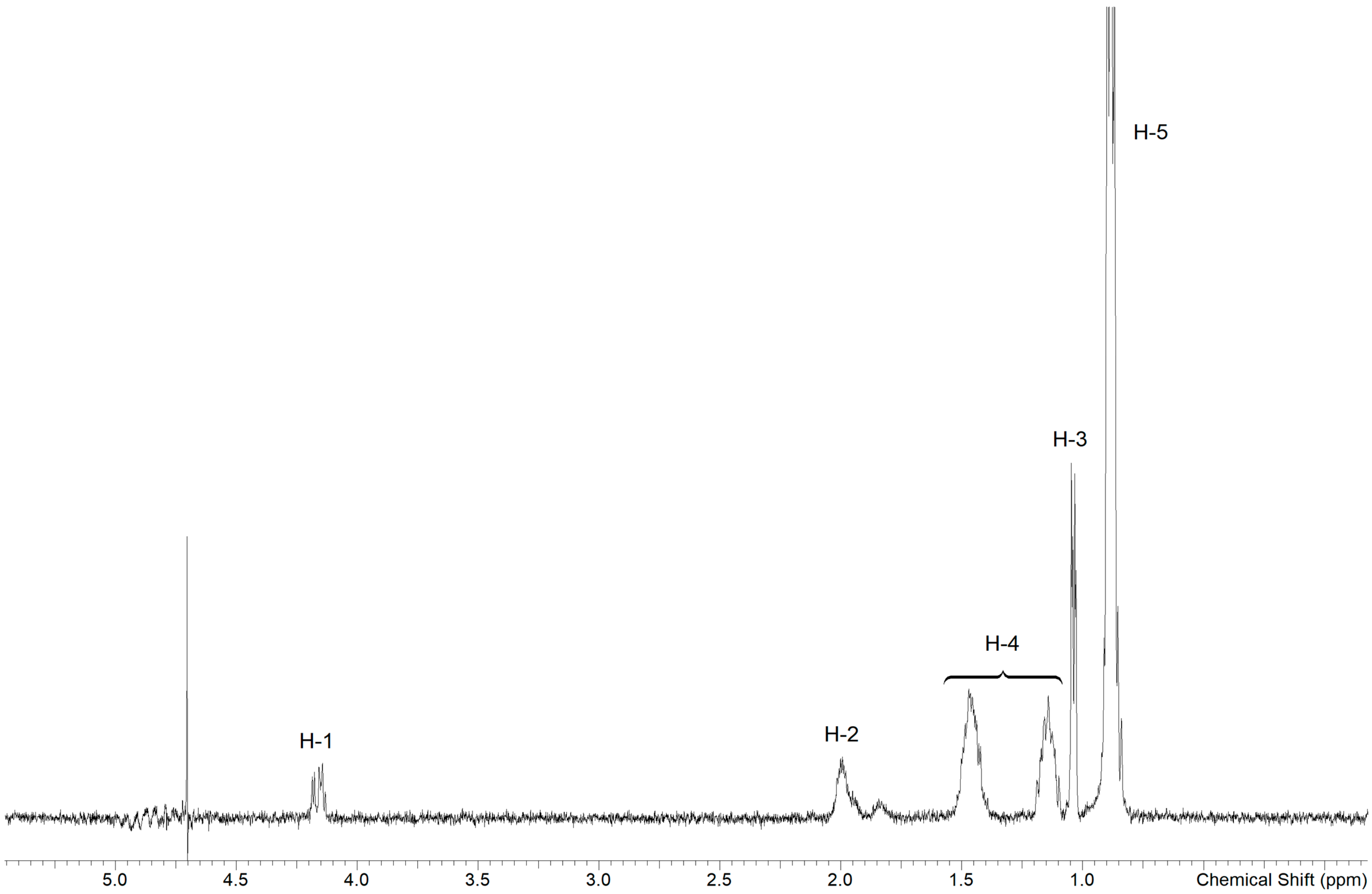

Figure S12. 1D selective TOCSY spectrum of miramide B (**2**)/ miramide C (**3**) measured in D_2O (500 MHz), irradiation at 0.88 ppm (H-2).

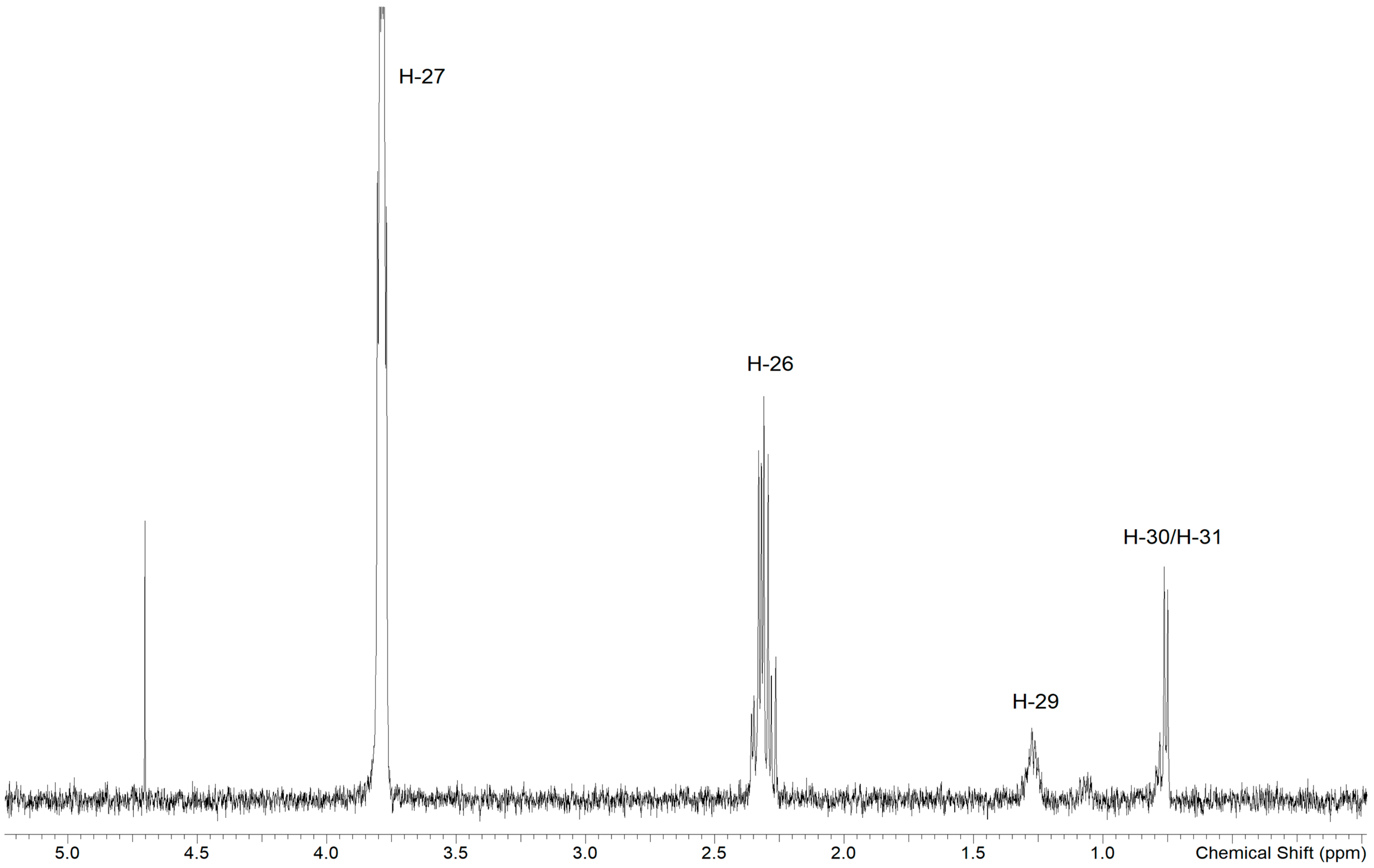

Figure S13. 1D selective TOCSY spectrum of miramide B (**2**)/ miramide C (**3**) measured in D_2O (500 MHz), irradiation at 3.79 ppm (H-27, B).

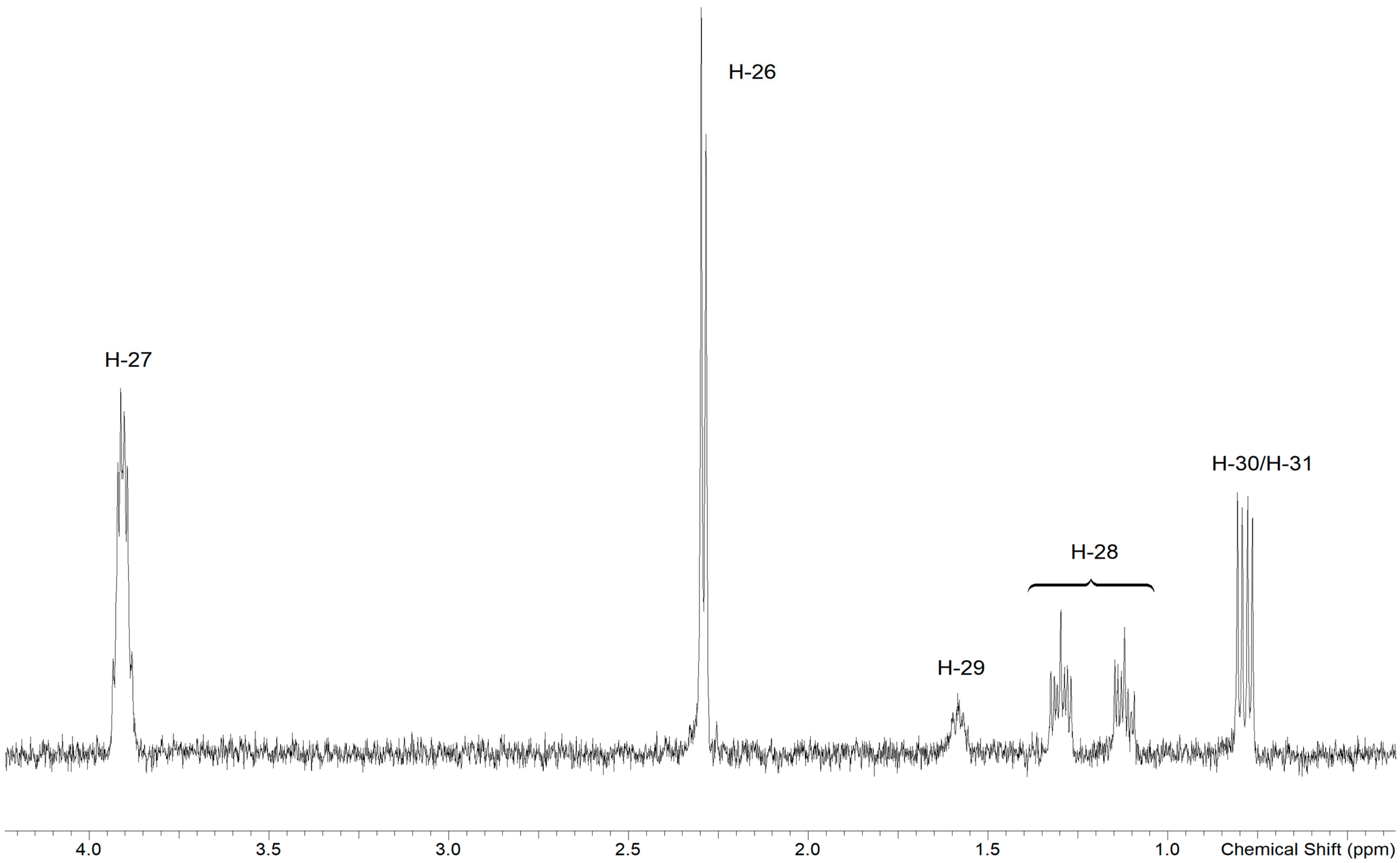

Figure S14. 1D selective TOCSY spectrum of miramide B (**2**)/ miramide C (**3**) measured in D_2O (500 MHz), irradiation at 3.91 ppm (H-27, C).

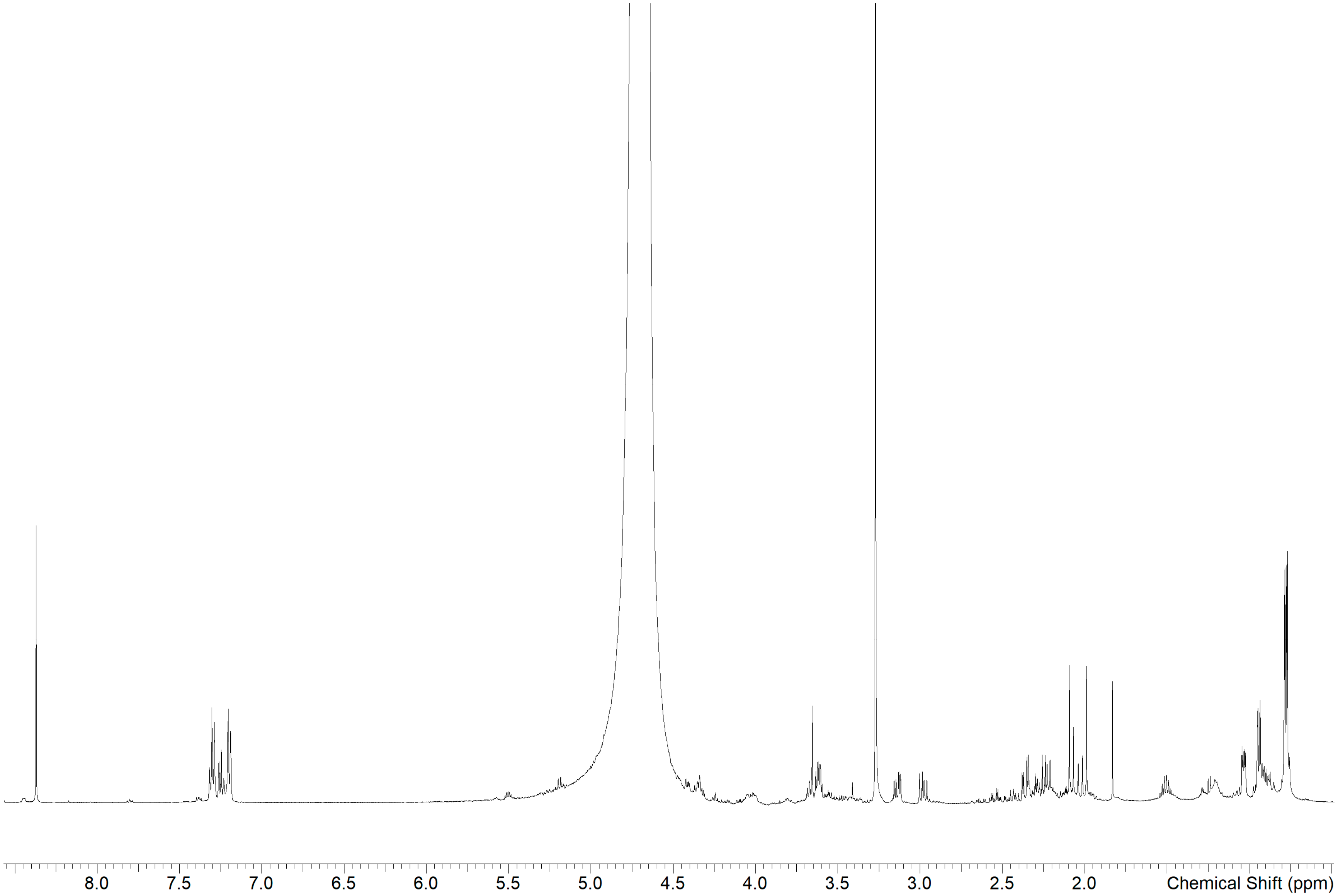

Figure S15. ^{1}H NMR spectrum of miramide D (**4**) measured in D_2O (500 MHz).

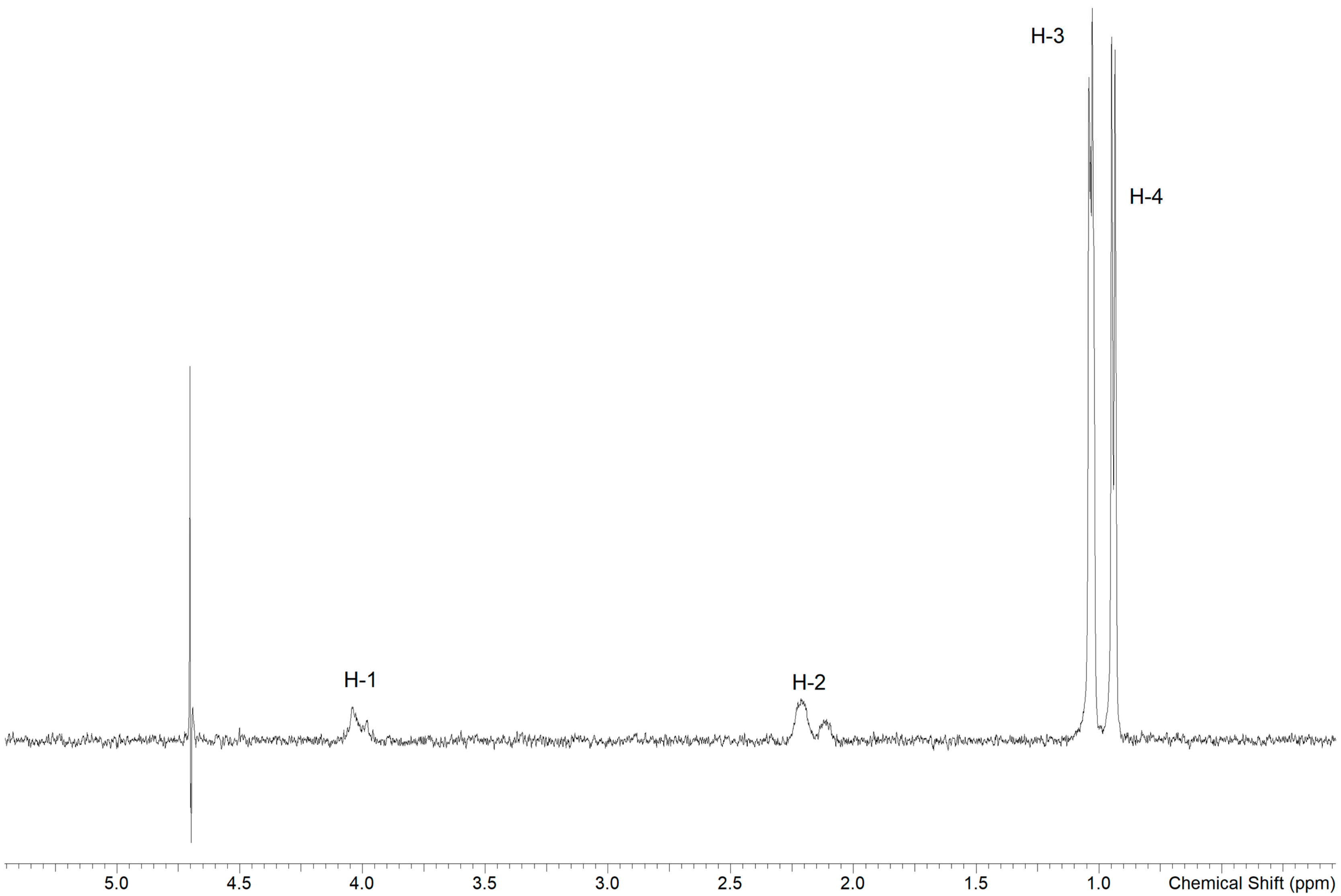

Figure S16. 1D selective TOCSY spectrum of miramide D (**4**) measured in D_2O (500 MHz), irradiation at 1.03 ppm (H-3).

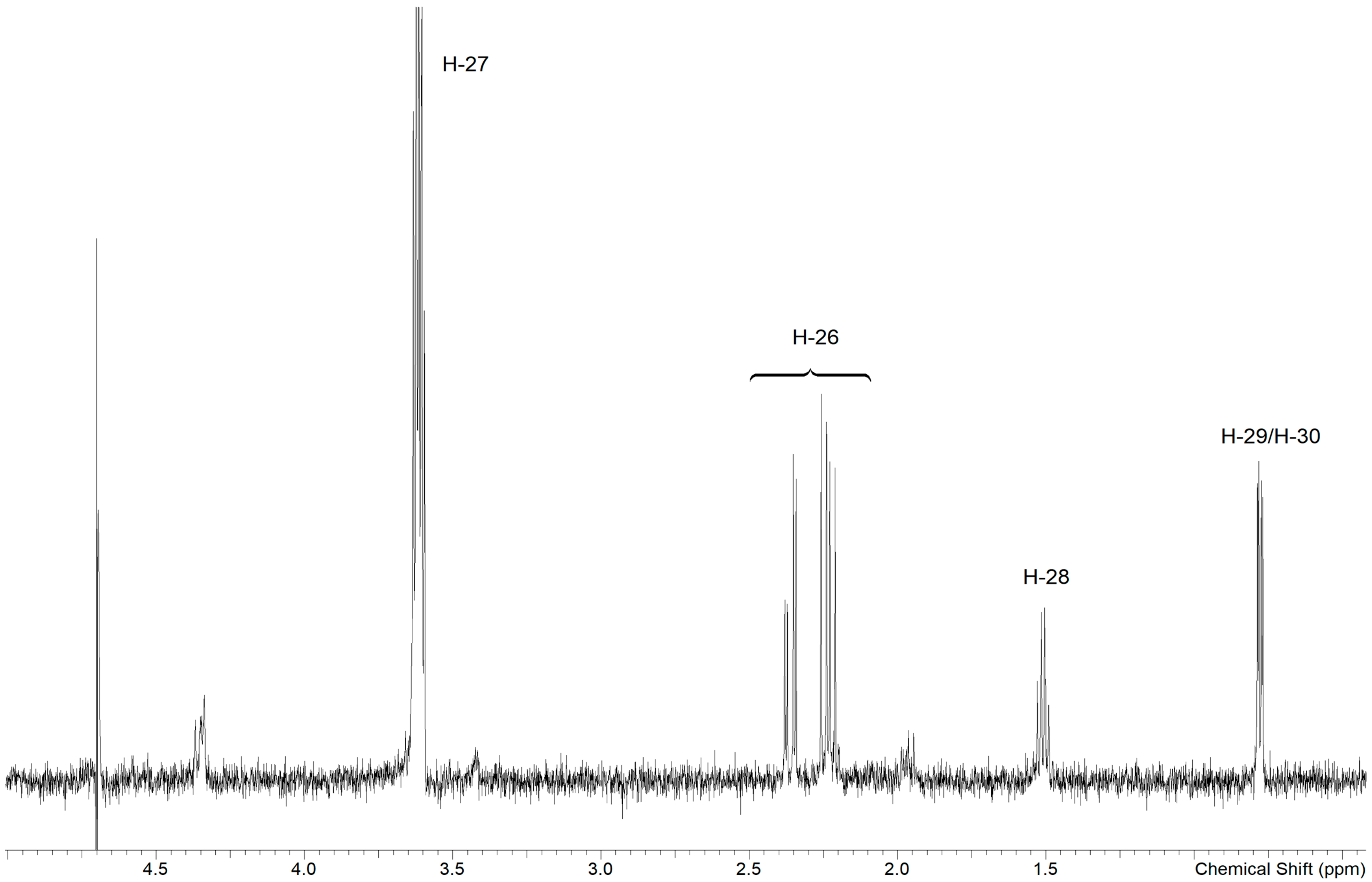

Figure S17. 1D selective TOCSY spectrum of miramide D (**4**) measured in D_2O (500 MHz), irradiation at 3.61 ppm (H-27).

References

1. Myronovskyi, M.; Rosenkränzer, B.; Nadmid, S.; Pujic, P.; Normand, P.; Luzhetskyy, A. Generation of a cluster-free Streptomyces albus chassis strains for improved heterologous expression of secondary metabolite clusters. *Metabolic engineering* **2018**, *49, doi:*10.1016/j.ymben.2018.09.004.
2. Flett, F.; Mersinias, V.; Smith, C.P. High efficiency intergeneric conjugal transfer of plasmid DNA from Escherichia coli to methyl DNA-restricting streptomycetes. *FEMS Microbiology Letters* **1997**, *155*, 223–229, doi:10.1016/S0378-1097(97)80014-6.
